# Inflammatory breast cancer microenvironment repertoire based on DNA methylation data deconvolution reveals actionable targets to enhance the treatment efficacy

**DOI:** 10.1186/s12967-024-05553-5

**Published:** 2024-08-05

**Authors:** Naiade Calanca, Flavia Lima Costa Faldoni, Cristiano Pádua Souza, Jeferson Santos Souza, Bianca Elen de Souza Alves, Milena Botelho Pereira Soares, Deysi Viviana Tenazoa Wong, Roberto César Pereira Lima-Junior, Fabio Albuquerque Marchi, Claudia Aparecida Rainho, Silvia Regina Rogatto

**Affiliations:** 1 Department of Clinical Genetics, University Hospital of Southern Denmark, Beriderbakken 4, Vejle, DK 7100 Denmark; 2 Department of Chemical and Biological Sciences, Institute of Biosciences, São Paulo State University (UNESP), Botucatu, SP 18618-689 Brazil; 3 Medical Oncology Department, Barretos Cancer Hospital, Pio XII Foundation, Barretos, SP 14784-400 Brazil; 4 Health Technology Institute, SENAI CIMATEC, Salvador, BA 41650-010 Brazil; 5 Department of Physiology and Pharmacology, Drug Research and Development Center (NPDM), Faculty of Medicine, Federal University of Ceará, Fortaleza, 60430-270 Brazil; 6 Gonçalo Moniz Institute, FIOCRUZ, Salvador, BA 40296-710 Brazil; 7 Department of Head and Neck Surgery, University of São Paulo Medical School, São Paulo, SP 05402-000 Brazil; 8 Center for Translational Research in Oncology, Cancer Institute of the State of São Paulo (ICESP), São Paulo, SP 01246-000 Brazil; 9 Institute of Regional Health Research, University of Southern Denmark, Odense, 5000 Denmark; 10 Botucatu Medical School Hospital, São Paulo State University (UNESP), Botucatu, SP Brazil

**Keywords:** Inflammatory breast cancer, DNA methylation, Tumor microenvironment, Endothelial cells, Immune markers, Epigenetic silencing, Deconvolution

## Abstract

**Background:**

Although the clinical signs of inflammatory breast cancer (IBC) resemble acute inflammation, the role played by infiltrating immune and stromal cells in this aggressive disease is uncharted. The tumor microenvironment (TME) presents molecular alterations, such as epimutations, prior to morphological abnormalities. These changes affect the distribution and the intricate communication between the TME components related to cancer prognosis and therapy response. Herein, we explored the global DNA methylation profile of IBC and surrounding tissues to estimate the microenvironment cellular composition and identify epigenetically dysregulated markers.

**Methods:**

We used the HiTIMED algorithm to deconvolve the bulk DNA methylation data of 24 IBC and six surrounding non-tumoral tissues (SNT) (GSE238092) and determine their cellular composition. The prognostic relevance of cell types infiltrating IBC and their relationship with clinicopathological variables were investigated. CD34 (endothelial cell marker) and CD68 (macrophage marker) immunofluorescence staining was evaluated in an independent set of 17 IBC and 16 non-IBC samples.

**Results:**

We found lower infiltration of endothelial, stromal, memory B, dendritic, and natural killer cells in IBC than in SNT samples. Higher endothelial cell (EC) and stromal cell content were related to better overall survival. EC proportions positively correlated with memory B and memory CD8^+^ T infiltration in IBC. Immune and EC markers exhibited distinct DNA methylation profiles between IBC and SNT samples, revealing hypermethylated regions mapped to six genes (*CD40*, *CD34*,* EMCN*,* HLA-G*,* PDPN*, and *TEK*). We identified significantly higher CD34 and CD68 protein expression in IBC compared to non-IBC.

**Conclusions:**

Our findings underscored cell subsets that distinguished patients with better survival and dysregulated markers potentially actionable through combinations of immunotherapy and epigenetic drugs.

**Supplementary Information:**

The online version contains supplementary material available at 10.1186/s12967-024-05553-5.

## Background

Inflammatory breast cancer (IBC) is characterized by inflammatory signs such as erythema, skin thickening (*peau d’orange* appearance), breast heat and swelling, and tumor emboli that frequently invade dermal-lymphatic vessels [1, 2]. Although up to 75% of IBC cases exhibit tumor emboli, their detection is not pathognomonic or required for the diagnosis, which is primarily based on clinical presentation [1]. The international consensus guidelines recommend a trimodal approach to treat most cases of IBC and inoperable non-metastatic locally advanced breast cancer, including systemic therapy, surgery, and radiation therapy [2]. Neoadjuvant chemotherapy is based on anthracycline-taxane regimens and is followed by a modified radical mastectomy, post-mastectomy radiation therapy, and adjuvant systemic therapy [3]. Despite improvements in multimodal therapy, IBC patients experience worse outcomes with low survival rates [4, 5].

Molecular signatures described in several tumor types have demonstrated their value for developing more effective and personalized treatments. Transcriptome analysis has revealed similar gene signatures between non-IBC and IBC cases [6–8]. Conversely, the stromal gene expression profile described in IBC differed from non-IBC [9]. However, these studies were performed using microarray platforms, which are limited by the amount of generated data.

The tumor microenvironment (TME) is a dynamic interface with mutual interactions between tumor and normal stromal cells. Accumulating evidence suggests a unique composition of different cell types in IBC compared to non-IBC. Using in vitro and in vivo (mice) pre-clinical experiments, Wolfe et al. (2016) showed a reciprocal interaction between mesenchymal stem cells and M2 macrophages to improve migration and high secretion of IL-6 in IBC cells [10]. Moreover, myeloid-derived dendritic cells [11], T cell receptor-activated CD4^+^ and CD8^+^ T cells were detected in clinical assays of IBC samples [12, 13]. A higher PDL1 expression was described in IBC compared with non-IBC clinical samples [12]. High PDL1 expression was associated with the degree of lymphocyte infiltrate, T-cell-specific, CD8 + T-cell-specific and B-cell-specific gene expression signatures, particularly cytotoxic T-cell response [12]. Increased CD4 + cell infiltration and its presence were correlated with better overall survival of IBC patients [13]. Interactions between IBC cells and the TME lead to a variety of pathway crosstalk (such as JAK-STAT, NF-κB, IL-6, TGFβ, and EGFR pathways) that can contribute to the IBC aggressiveness [4].

Immune checkpoint inhibitors are successful immunotherapy strategies targeting co-inhibitory immune checkpoints such as PD1/PDL1 and CTLA4 and have been tested in several cancers [14]. PDL1 expression is higher in IBC than in non-IBC at mRNA and protein expression levels [15], and a positive correlation was found with complete pathological response to chemotherapy [12]. Exploration of additional actionable targets is essential, although the low number of cells has limited the characterization of immune TME in IBC samples.

Single-cell technologies allow the interrogation of heterogenous tumor samples to elucidate the contribution of different cell types. However, the high costs and protocol-specific bias leading to inaccurate representation of cell types are still limiting factors for these analyses [16]. In this sense, exploring widely available bulk data to infer the TME repertoire is more cost-effective. Deconvolution analysis based on transcriptome data has been described for several tumor types, including IBC [17–20]. At least 20 deconvolution tools based on transcriptomics data are available [21]. Zhang et al. (2023) performed deconvolution analysis in cDNA microarray data from 20 IBC, 20 non-IBC, and five normal breast tissue samples using CIBERSORTx [20]. This study showed that memory B cells are enriched, while activated mast cells and eosinophils are diminished in IBC compared to normal samples. The results also indicated that activated mast cells and follicular helper T cells are reduced, while M2 macrophages are increased in IBC compared to non-IBC. Bertucci et al. (2021), applying CIBERSORT analysis based on gene expression profiles of 137 IBC compared to 252 non-IBC samples, found enriched M1 macrophages, γδ T-cells, and memory B cells infiltration, higher expression of tertiary lymphoid structures and T cell-inflamed signature [18]. The authors also identified several actionable immune genes overexpressed (*HAVCR2/TIM3*,* CD27*,* CD70*,* CTLA4*,* ICOS*,* IDO1*,* LAG3*,* PDCD1*,* TNFRSF9*,* PVRIG*,* CD274/PDL1*, and *TIGIT*) in IBC. While the number of transcripts is highly variable and might not be proportional to the number of cells, differentially methylated regions are usually constant across individuals within cell types [22]. Since each CG dinucleotide has only two possible states (methylated or not) and DNA methylation is consistent throughout cellular differentiation [23], methylome data is especially suited for deconvolution. To our knowledge, no previous study with IBC samples has explored DNA methylation data to identify the contribution of different cell types using deconvolution analysis.

Herein, we used the Hierarchical Tumor Immune Microenvironment Deconvolution (HiTIMED) tool based on DNA methylation [24], which allowed the analysis of 17 cell types (angiogenic, immune, and tumor components) of the IBC microenvironment. HiTIMED employs tumor-specific signatures derived from primary cancer cells and enhances cell projection accuracy by using a hierarchical model to deconvolve the TME. Based on this analysis, we identified epigenetically dysregulated markers associated with the tumor-infiltrating cell types.

## Materials and methods

### Samples and data acquisition

Genome-wide DNA methylation profiling was previously performed in 24 biopsies of inflammatory breast carcinomas (IBC) and six surrounding non-tumoral tissue (SNT) samples matched to the IBC samples (discovery cohort) using the Infinium MethylationEPIC BeadChip array (Illumina, San Diego, CA, USA). Data was deposited in the Gene Expression Omnibus (GEO) database (GSE238092) [25]. More information about DNA methylation data generation and analysis is available in Additional file 1 (Supplementary Methods). Gene expression data (microarray) of 20 IBC and five normal breast tissue samples publicly available on GEO (GSE45581) was also explored [8]. The study was conducted according to the guidelines of the Declaration of Helsinki, approved by the Human Research Ethics Committee of Barretos Cancer Hospital (CEP# 37779220.5.1001.5437). Informed consent was obtained from all subjects involved in the study.

The DNA methylation data analyzed in this study was generated using IBC specimens collected before the patients’ treatment. In six cases, we also collected SNT samples, which were histologically confirmed as not having tumor cells. Among the IBC samples, ten were triple negative (TN), 11 were estrogen receptor (ER)/ progesterone receptor (PR)-positive, and three were human epidermal growth factor receptor type 2 (HER2)-positive (Table S1, Additional file 1). A set of 17 IBC and 16 non-IBC formalin-fixed paraffin-embedded (FFPE) tissue samples (validation cohort) was evaluated by immunofluorescence staining (Table S1, Additional file 1). These samples were paired according to clinical stage.

A flowchart summarizing the study design and the experimental methods and analyses applied to each cohort is depicted in Fig. S1 (Additional file 2).

### Deconvolution of bulk tumor samples

To predict the relative fraction of cell types from the bulk tumor samples, we used the HiTIMED deconvolution method. The methylation beta matrix from the discovery cohort (24 IBC and six SNT) was used to estimate the proportions of 17 cell types with R software (v. 4.3.1) and HiTIMED algorithm [24]. HiTIMED contains six hierarchical layers that use reference libraries to estimate cell proportions in 20 tumor types, which allows the user to select tumor site and layer. The parameters tumor type “BRCA” (breast invasive carcinoma) and the layer of deconvolution “6” were selected for the analysis. Partitioning around medoids (PAM) clustering was performed with cluster R package (v 2.1.4) to classify immune hot and immune cold tumors using the HiTIMED results.

### Immune markers selection

Using the DNA methylation dataset from the discovery cohort, we explored 73 markers and secreted factors related to the altered immune cell populations. According to the Illumina manifest, 1,549 probes are mapped to these genes (Table S2, Additional file 3).

We also investigated the DNA methylation levels of 50 genes encoding co-stimulatory receptors, adhesion molecules, and chemokines expressed by endothelial cells (ECs), as previously reported [26, 27]. According to the Illumina manifest, 1,556 probes are mapped to these genes (Table S3, Additional file 3). To further characterize these EC markers, we calculated the differences in gene expression between IBC and normal breast tissue samples (data normalized using limma R/Bioconductor package - version 3.58.1) using external cDNA microarray data (GSE45581). Gene expression was calculated as an average when multiple probes were mapped to the same gene.

### CD34 and CD68 immunofluorescence

The FFPE tumor tissues (IBC and breast cancer other than IBC) obtained from the validation cohort were deparaffinized in xylene, hydrated through a graded ethanol series, and washed under tap water and phosphate-buffered saline (PBS). The antigenic recovery was performed using 0.1 M (pH 6.0) sodium citrate buffer at 95 °C for 30 min. Permeabilization was performed using 0.1% Triton X-100 (Sigma-Aldrich, St. Louis, MO, USA). The slides were incubated with a solution containing 0.3 M glycine (Sigma-Aldrich, St. Louis, MO, USA) and 5% bovine serum albumin (Sigma-Aldrich, St. Louis, MO, USA) for 1 h at room temperature to block non-specific binding sites. The tissue sections were incubated overnight, at 2–8 °C, with the monoclonal primary antibody mouse anti-CD34 (1:100, Cell Signaling Technology, Massachusetts, USA) or monoclonal primary antibody rabbit anti-CD68 (1:100, Sigma-Aldrich, Merck, USA). After primary antibody incubation, the slides were washed (3x in PBS for 5 min each) and incubated with the secondary antibody goat anti-mouse IgG Alexa Fluor 568 (1:400, Thermo Fisher Scientific, Waltham, MA, USA) for 1.5 h at room temperature. Next, the slides were washed (3x in PBS for 5 min each), and tissues were exposed to a 0.002% 4,6’-diamidino-2-phenylindole (DAPI)/ PBS solution (Thermo Fisher Scientific) for 30 min to label the cell nuclei. The slides were washed (3x in PBS for 5 min) again and mounted with ProLong Gold Antifade Mountant (Thermo Fisher Scientific). Coverslips were applied and sealed. The photomicrographs were obtained using an immunofluorescence microscope (Agilent BioTek Cytation, Santa Clara, CA, USA) with standardization of master gain and digital offset for analysis. The obtained images were analyzed using image software (Fiji Image J, National Institutes of Health, Washington, DC, USA). The fluorescent area was quantified by differentiating the fluorescent pixels related to red fluorescence (Alexa Fluor 594) and the blue staining to identify cell nuclei marked with DAPI. Detection color thresholds were established and standardized across all quantifications. The obtained data was expressed as the fluorescent area by comparing the fluorescence intensity of the target marker with DAPI (100%).

### Statistical analyses and data visualization

Statistical analyses were performed using GraphPad Prism (GraphPad Software, version 8.3.0, USA) and R software (version 4.3.1). The Mann–Whitney U-test was applied to compare cell proportions between IBC and SNT or triple-negative breast carcinoma (TN) and non-TN samples (non-parametric data). Kaplan–Meier method and the log-rank (Mantel-Cox) test were used for univariate survival analysis and curve comparisons, respectively. We considered overall survival (OS) as the interval between the date of IBC diagnosis and death from any cause. Patients were stratified into two groups (high and low cell proportion) by the median fractions estimated using HiTIMED. Multiple logistic regression analysis was performed using the ‘stats’ R package to determine the clinicopathological variables correlated with EC and stromal cell content (high *versus* low) in IBC samples. The heatmaps were generated using Morpheus web-based tool (https://software.broadinstitute.org/morpheus/, last accessed on 29 October 2023) and finalized in Inkscape v1.0.2. Euclidean distance and complete linkage were used for unsupervised hierarchical clustering. The plots depicting gene expression levels were generated using ggplot2 R package.

## Results

**Fig. 1:**
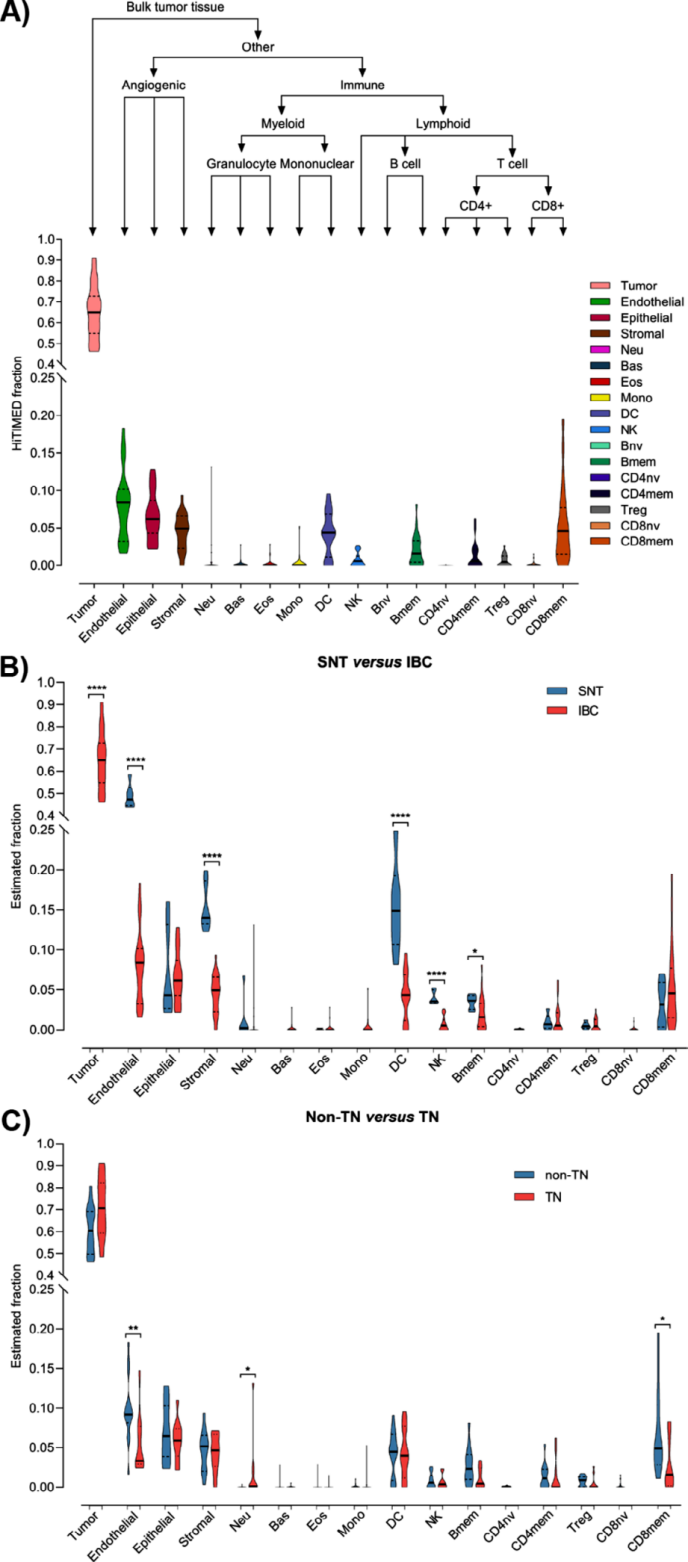
Deconvolution of inflammatory breast cancer (IBC) based on DNA methylation data using HiTIMED. **A**) Structure of the HiTIMED layers of deconvolution and distribution of cell composition in our dataset of 24 IBC samples. The violin plots depict the differences between (**B**) 24 IBCs and six surrounding non-tumoral tissues (SNT), and (**C**) ten triple-negative (TN) and 14 non-TN IBC samples for 16 detected cell types. Naïve B cells were not detected in any samples. The statistical difference was determined by the Mann–Whitney test (* *p* ≤ 0.05, ** *p* ≤ 0.01, **** *p* ≤ 0.0001). The solid line represents the median, and the dashed lines represent the first and third quartiles in the violin plots. Bas: basophil; Bmem: memory B cell; CD4mem: memory CD4 + T cell; CD4nv: naïve CD4 + T cell; CD8mem: memory CD8 + T cell; CD8nv: naïve CD8 + T cell; DC: dendritic cell; Eos: eosinophil; Mono: monocyte; Neu: neutrophil; NK: natural killer cell; Treg: regulatory T cell

The proportion of 17 cell types was estimated by HiTIMED for samples from the discovery cohort (Fig. S2, Additional file 4) and the content of tumor cells represented, on average, 65% of the IBC samples (Fig. 1A). ECs, stromal cells, memory B cells, dendritic cells (DC), and natural killer (NK) cells showed lower infiltration in IBC compared to SNT samples (Fig. 1B). The comparison between TN and non-TN samples revealed that ECs and memory CD8^+^ T cells infiltration is reduced, while neutrophils infiltration is enhanced in TN (Fig. 1C).

**Fig. 2:**
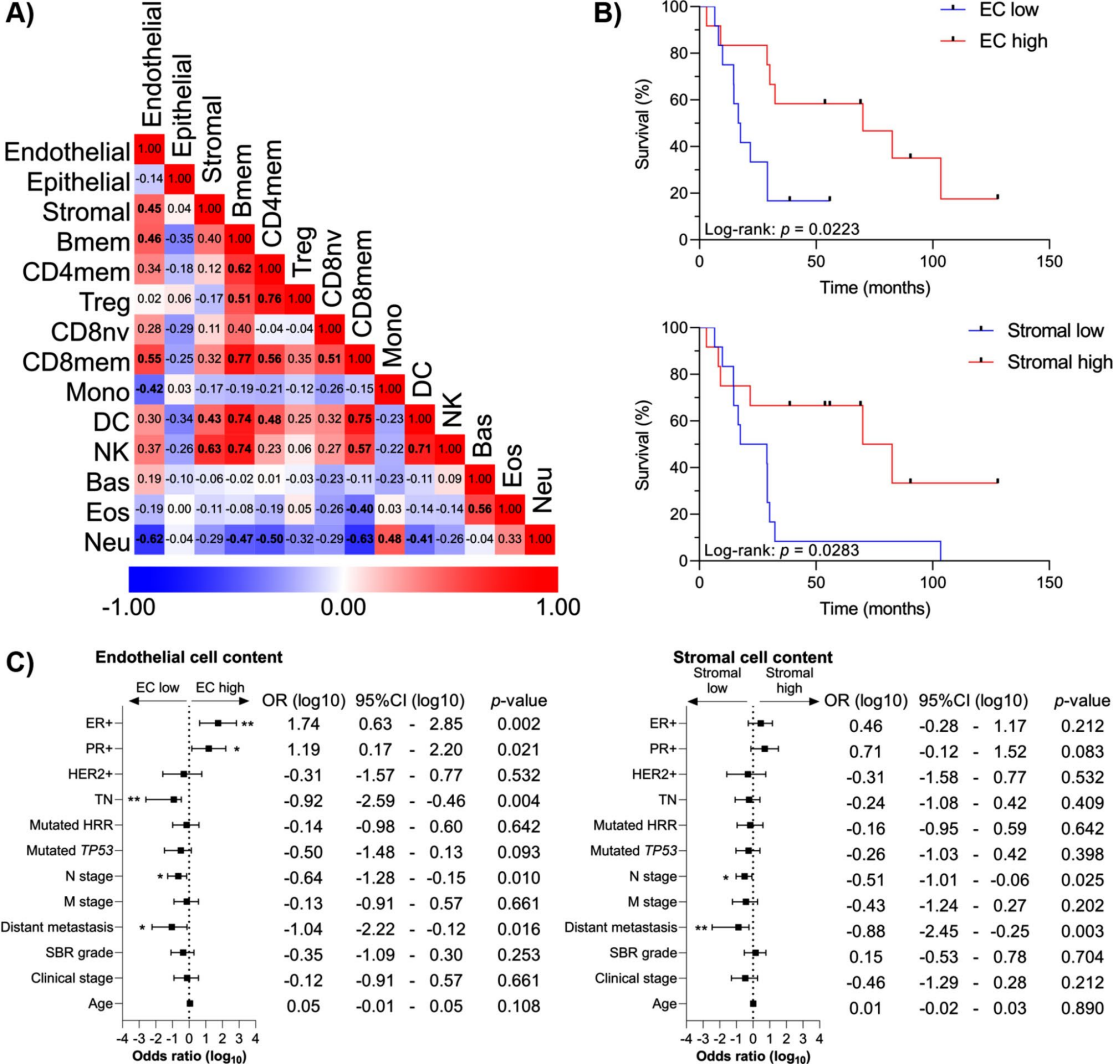
Estimated cell fractions (HiTIMED) and clinicopathological variables in inflammatory breast cancer (IBC). (**A**) Matrix showing the pairwise relationship between immune and angiogenic cell fractions estimated by HiTIMED. The numerical values represent Spearman’s rank correlation coefficients, and they are color-coded according to the scale below the matrix. Statistically significant correlations are indicated in bold (*p* < 0.05). (**B**) Kaplan–Meier curves showing the impact of endothelial and stromal cells on the survival of 24 IBC patients (*p*-values from Log-rank Test). The cutoffs for survival analysis were determined using median values of the estimated cell fractions. (**C**) Forest plots showing clinicopathological variables correlated with endothelial and stromal cell content (high *versus* low) in IBC. The error bars represent a 95% confidence interval (CI) with the odds ratio (OR) result displayed as a plotted box. * *p* ≤ 0.05, ** *p* ≤ 0.01. Bas: basophil; Bmem: memory B cell; CD4mem: memory CD4 + T cell; CD4nv: naïve CD4 + T cell; CD8mem: memory CD8 + T cell; CD8nv: naïve CD8 + T cell; DC: dendritic cell; EC: endothelial cell; Eos: eosinophil; Mono: monocyte; Neu: neutrophil; NK: natural killer cell; Treg: regulatory T cell

We investigated the relationships between the immune and angiogenic cells predicted in the microenvironment of 24 IBC samples by calculating the correlation based on the estimated cell fractions (Fig. 2A). Twenty-six out of 91 pairs of immune and angiogenic cell types showed significant correlations (Spearman’s rank correlation, *p* < 0.05). Stromal cells were positively correlated to DCs and NK cells (*R* = 0.43 and 0.63, respectively). ECs were positively correlated to stromal, memory B, and memory CD8^+^ T cells (*R* = 0.45, 0.46, and 0.55, respectively). Monocytes and neutrophils displayed inverse correlations with ECs (*R* = − 0.42 and − 0.62).

Clinicopathological variables or estimated cell fractions were correlated with patients’ survival (Table 1). The clinical stage (*p* < 0.0001), distant metastasis (*p* = 0.003), M stage (*p* < 0.0001), and N stage (*p* = 0.0017) had a significant impact on OS (univariate analysis). Interestingly, we observed that IBC cases with higher EC and stromal cell proportions presented increased OS (*p* = 0.0223 and *p* = 0.0283, respectively) (Fig. 2B; Table 1). We verified the distribution of EC and stromal cell clusters (high *versus* low) according to the presence of metastasis (M0 *versus* M1) and clinical stage (stages IIIB + IIIC *versus* stage IV) using Fisher’s exact test and did not find statistically significant differences between non-metastatic and metastatic patients or stages III and IV (Fig. S3, Additional file 5). Also, the tumor cell content does not affect the patients’ survival (*p* = 0.9756) (Fig. S4, Additional file 6). Among the six variables (clinical stage, distant metastasis, M stage, N stage, EC, and stromal cell proportions) that significantly impacted IBC patients’ OS according to the Kaplan-Meier curves and log-rank test (Table 1), five were also significant according to the univariate Cox regression analysis: clinical stage, distant metastasis, M stage, EC, and stromal cell content (Table S4, Additional file 1). Our limited sample size precluded the multivariate analysis to determine the value of these factors as independent survival predictors. Multiple logistic regression was performed to determine which variables correlated with EC and stromal cell content. Advanced N stage and distant metastasis were more frequently observed in EC- and stromal cell-low than in EC- and stromal cell-high groups (Fig. 2C). Additionally, TN cases were more common in the EC-low group, while the proportion of ER-positive and PR-positive tumors was higher in the EC-high group.

**Table 1:** Kaplan-Meier survival analysis based on clinicopathological variables and estimated cell fractions (HiTIMED)

Variable	Number of cases	Median survival (months)	*p*-value (log-rank test)
Age (years)			
≤ 55	14	19.23	0.2669
> 55	10	51.21	
Clinical stage			
IIIB + IIIC	15	70.03	**< 0.0001**
IV	9	14.66	
SBR grade			
1 + 2	16	31.26	0.3027
3	8	25.48	
Distant metastasis			
Presence	15	21.80	**0.003**
Absence	9	103.48	
M stage			
M0	15	70.03	**< 0.0001**
M1	9	14.66	
N stage			
N0 + N1	9	103.48	**0.0017**
N2 + N3	15	17.51	
*TP53* mutation*			
Positive	10	15.69	0.2074
Negative	14	51.21	
HRR genes mutation*			
Positive	7	29.15	0.4183
Negative	17	29.15	
Triple negative			
Yes	10	19.23	0.1114
No	14	51.21	
ER			
Positive	11	70.03	0.1642
Negative	13	17.51	
PR			
Positive	8	51.21	0.8518
Negative	16	25.36	
HER2			
Positive	3	17.51	0.9236
Negative	21	29.15	
EC cluster			
Low	12	17.08	**0.0223**
High	12	70.03	
Stromal cluster			
Low	12	23.21	**0.0283**
High	12	76.31	

SBR: Scarff-Bloom-Richardson grading system; HRR: homologous recombination repair; ER: estrogen receptor; PR: progesterone receptor; HER2: human epidermal growth factor receptor type 2; EC: endothelial cell; in bold: *p*-value < 0.05. *Only pathogenic and likely pathogenic variants were considered

**Fig. 3:**
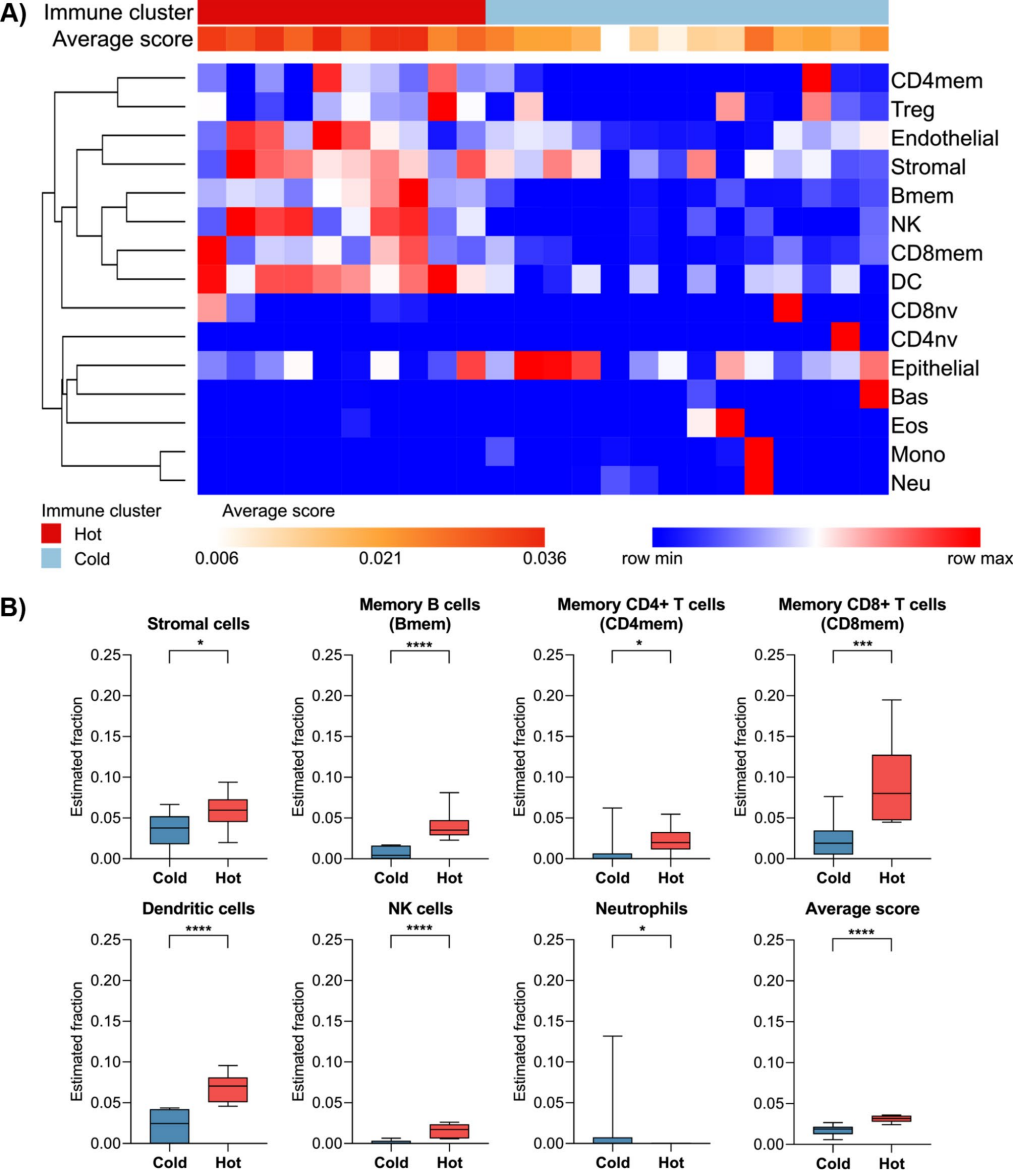
Classification of inflammatory breast cancer (IBC) samples into immune hot and cold. (**A**) Partitioning around medoids (PAM) clustering using the HiTIMED estimated cellular fractions revealed two clusters with different immune and angiogenic cell type distributions. The average score was calculated for each IBC sample considering the estimated fraction of 15 cell types. Rows were clustered based on the Euclidean distance of the estimated fractions. (**B**) Boxplots of cell types based on the two clusters (hot and cold) derived from IBC samples. The estimated fraction of each cell type in hot *versus* cold tumors was represented only for significant differences (*p* < 0.05, Mann-Whitney test). *p ≤ 0.05, ***p ≤ 0.001, ****p ≤ 0.0001

We performed PAM clustering analysis (k = 2) on the estimated cellular fractions of IBC cases to define subgroups based on infiltration patterns. Two clusters (immune cold and immune hot) showed markedly different distributions of multiple cell types, most notably stromal cells, memory CD4^+^ T-, CD8^+^ T-, and B-lymphocytes, DC, NK cells, and neutrophils. Immune hot specimens also presented a higher average score (the average score of 15 immune and angiogenic cell types calculated for each IBC sample) (Fig. 3A-B).

**Fig. 4:**
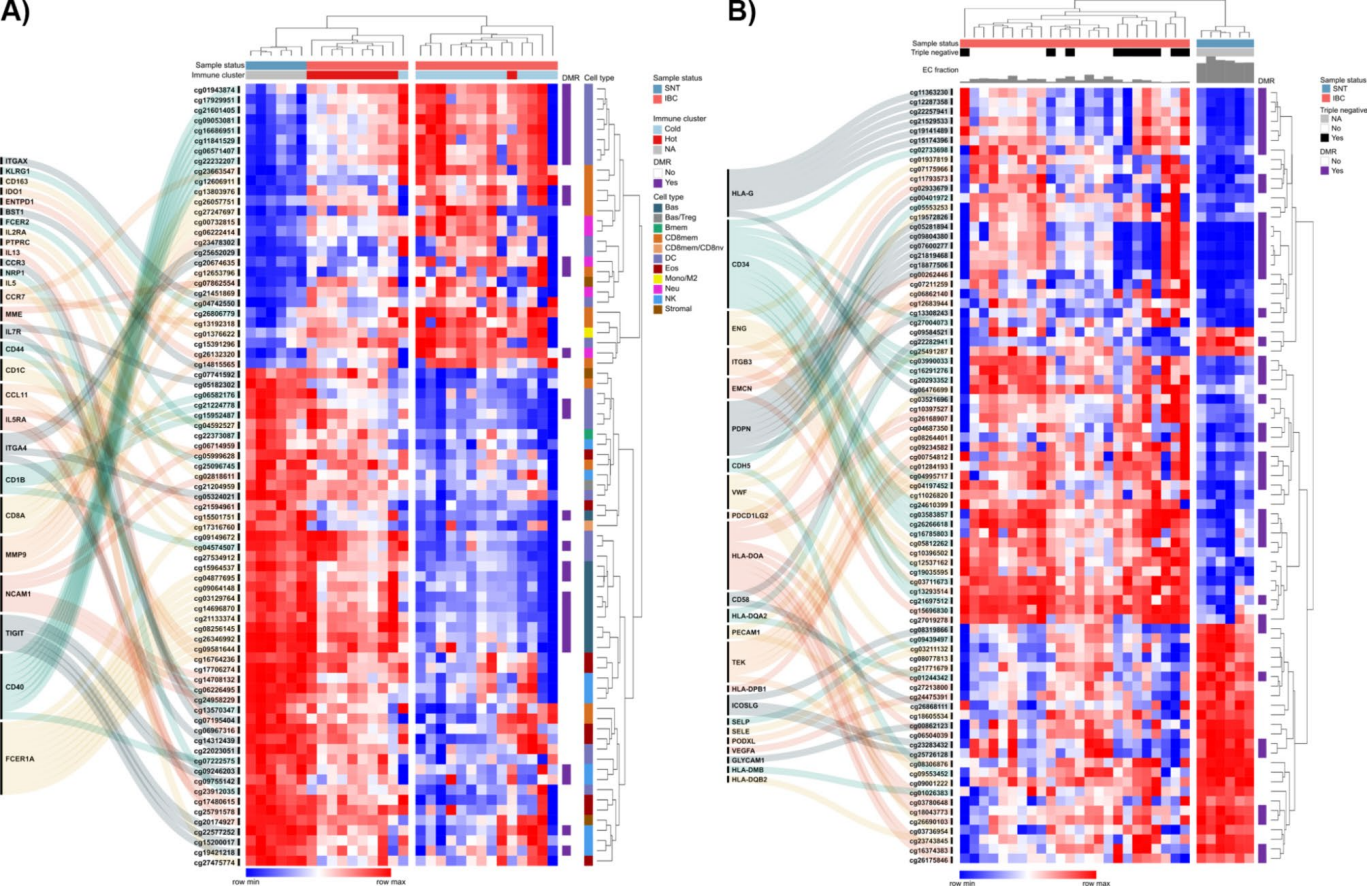
DNA methylation profile of immune and endothelial cell (EC) markers in inflammatory breast cancer (IBC). Heatmaps depicting the differentially methylated probes (DMPs) mapped to genes encoding (**A**) immune and (**B**) EC markers and secreted factors in samples from the discovery cohort (IBC in pink and SNT in blue). Rows and columns were clustered based on the Euclidean distance of the normalized DNA methylation levels. The purple bar indicates DMPs associated with differentially methylated regions (DMRs). Bas: basophil; Bmem: memory B cell; CD8mem: memory CD8 + T cell; CD8nv: naïve CD8 + T cell; DC: dendritic cell; EC: endothelial cell; Eos: eosinophil; Mono: monocyte; Neu: neutrophil; NK: natural killer cell; SNT: surrounding non-tumoral tissue; Treg: regulatory T cell

The DNA methylation dataset (GSE238092) comprised 46,908 differentially methylated probes (DMPs) detected in 24 IBC compared to six SNT samples, including 4,369 differentially methylated regions (DMRs) associated with 3,255 genes. We explored this set of probes focusing on protein-coding genes relevant to the immune cell populations. Seventy-seven DMPs were mapped to 28 genes encoding immune markers or secreted factors: *BST1*,* CCL11*,* CCR3*,* CCR7*,* CD163*,* CD1B*,* CD1C*,* CD40*,* CD44*,* CD8A*,* ENTPD1*,* FCER1A*,* FCER2*,* IDO1*,* IL13*,* IL2RA*,* IL5*,* IL5RA*,* IL7R*,* ITGA4*,* ITGAX*,* KLRG1*,* MME*,* MMP9*,* NCAM1*,* NRP1*,* PTPRC*, and *TIGIT*. Unsupervised clustering analysis based on normalized DNA methylation levels of this set of DMPs revealed two clusters: one including almost exclusively immune cold samples and the other mainly SNT and immune hot samples (Fig. 4A). Three genes encoding immune markers were associated with hypermethylated DMRs (*CD40* promoter, and *CD8A* and *MMP9* gene bodies), and three were associated with hypomethylated DMRs (*CD1B* gene body, *FCER1A* promoter, and *TIGIT* promoter/gene body) in IBC.

Since deconvolution analysis revealed that ECs were less represented in IBC compared to SNT samples, we investigated if the DNA methylation levels of EC markers would support this result. Eighty-one DMPs were mapped to 23 genes encoding EC surface proteins or secreted factors: *CD34*,* CD58*,* CDH5*,* EMCN*,* ENG*,* GLYCAM1*,* HLA-DMB*,* HLA-DOA*,* HLA-DPB1*,* HLA-DQA2*,* HLA-DQB2*,* HLA-G*,* ICOSLG*,* ITGB3*,* PDCD1LG2*,* PDPN*,* PECAM1*,* PODXL*,* SELE*,* SELP*,* TEK*,* VEGFA*, and *VWF*. These DMPs distinguished two clusters comprising IBC and SNT samples (Fig. 4B). Also, most DMPs exhibited increased methylation levels in IBC compared to SNT. Among these genes, five (*CD34*,* EMCN*,* HLA-G*,* PDPN*, and *TEK*) showed hypermethylated DMRs mapped to promoter regions, and three exhibited hypomethylated DMRs (gene body/3` UTR of *HLA-DOA*, and gene bodies of *HLA-DQA2* and *ICOSLG*) in IBC. Among the five genes with hypermethylated promoters, only *EMCN* presented a trend of downregulation in IBC (*p* = 0.07096) according to the external transcriptomic dataset (GSE45581). Moreover, *HLA-G* expression levels were increased (*p* = 0.01536), and *PDPN* presented a trend to upregulation (*p* = 0.06015) in IBC (Fig. S5, Additional file 7).

**Fig. 5:**
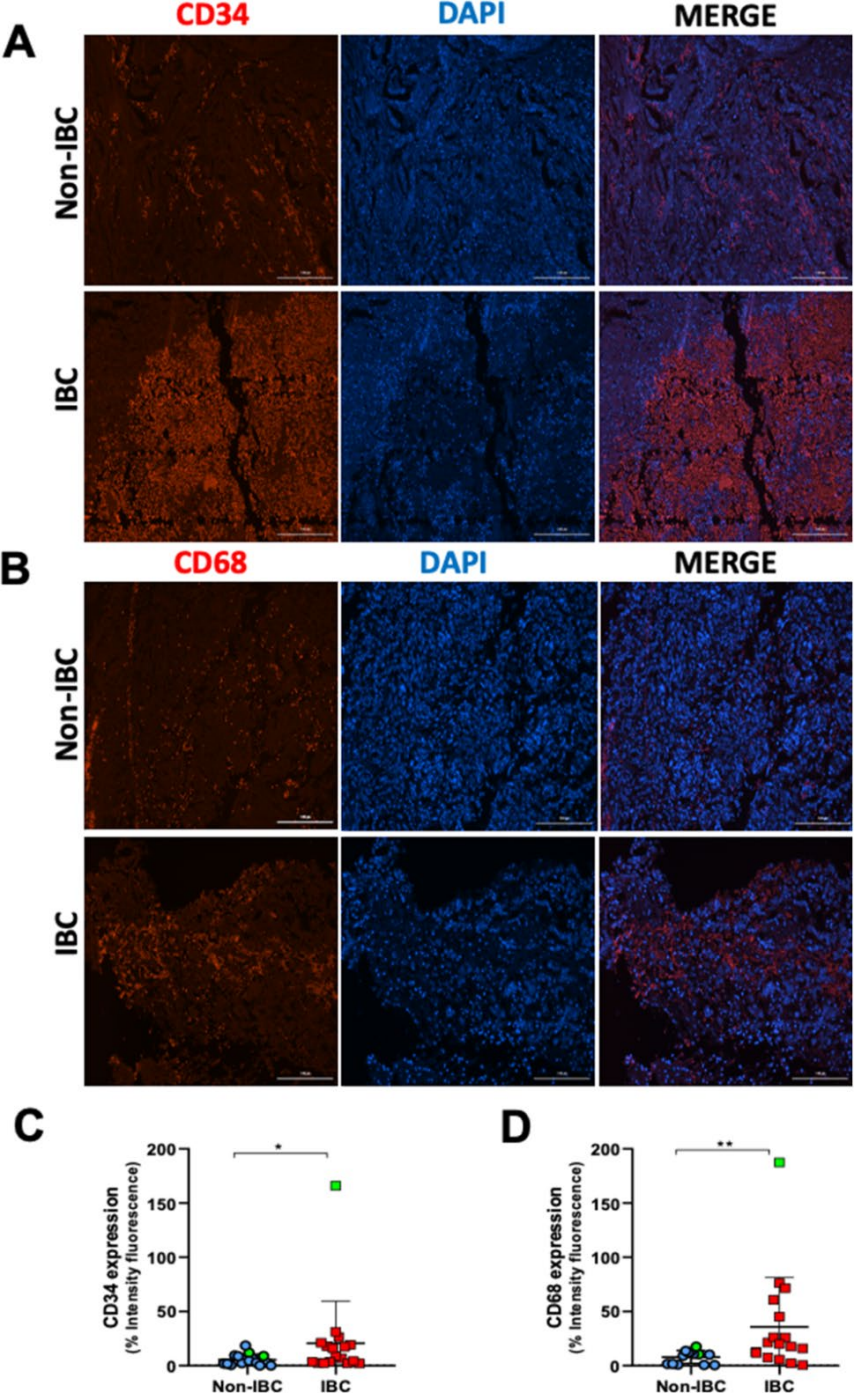
Molecular phenotyping for CD34 and CD68 in inflammatory breast cancer (IBC) and non-IBC samples. Immunofluorescence staining for (**A**) endothelial marker CD34 and (**B**) macrophage marker CD68. The scale bar represents 100 μm (Magnification 200 ×). The quantification of the percentage of fluorescence intensity showed significantly higher (**C**) CD34 and (**D**) CD68 protein expression in IBC (red dots) compared to non-IBC (blue dots) samples. Green dots: triple-negative tumors. The values are expressed as the means ± SD. Data were analyzed by the Mann-Whitney test. Statistical significance was set at * *p* < 0.05, ** *p* < 0.01

The molecular phenotype analysis using immunofluorescence assays showed a significant difference between IBC and non-IBC samples for CD34 (*p* < 0.05) and CD68 (*p* < 0.01), EC and macrophage markers, respectively (Fig. 5). CD34 and CD68 exhibited higher protein expression in IBC compared to non-IBC samples from the validation cohort (Fig. 5C-D). As demonstrated in Table S1 (Additional file 1), the comparison with the discovery set revealed that both cohorts are similar regarding most clinicopathological characteristics, including age, family history of cancer, clinical stage, histological grade, and presence of distant metastasis. These results support the coherence of evaluating the expression of cell-type-specific markers in the validation set to confirm the immune and EC content identified in the discovery set.

The survival analysis performed in the validation dataset based on clinicopathological variables and CD34 and CD68 protein expression is detailed in Table S5 (Additional file 1). The validation cohort exhibited significant differences in OS according to clinical stage, histological grade, presence of distant metastasis, M stage, TN breast tumors, and ER status (*p* < 0.05). Neither CD34 nor CD68 expression levels impacted OS.

## Discussion

It is still poorly understood whether inflammation contributes to tumorigenesis or disease aggressiveness, which is frequently described in IBC. Despite the clinical signs, which resemble the presence of acute inflammation, IBCs produce inflammatory cytokines (such as IFNG, TNF, IL1A, IL1B, and IL8) at similar levels to other breast cancer subtypes, while the host inflammatory cell infiltration is low in the IBC stroma [28, 29]. In this study, we explored differentially methylated probes and regions mapped to protein-coding genes relevant to immune and angiogenic cell populations from IBC microenvironment. DNA methylation plays a crucial role in the function of immune cells, including their maturation, polarization, and differentiation, and in tumor immune evasion [30]. In this scenario, epigenetic modifications emerge as indicators of the TME immune status and potential targets to improve immunotherapy responsiveness.

Cancer cells, their immediate environment, and surroundings were investigated employing the novel deconvolution algorithm HiTIMED, which can resolve 17 cell types in the TME using DNA methylation data [24]. HiTIMED includes cell types, such as DCs, that are prominent in shaping IBC microenvironment behavior [4]. Another advantage of HiTIMED compared with other deconvolution tools is that the algorithm uses tumor-specific signatures derived from primary breast cancer cells.

We observed that memory B, endothelial, stromal, NK cells, and DCs are diminished in IBC compared to SNT samples. Two previous studies reported a memory B cell enrichment in IBC using transcriptome-based deconvolution; one of them compared IBC to non-IBC samples [18, 20]. In our study, we deconvolved DNA methylation data and used non-tumoral samples as controls. Recently, Hannon et al. (2024) failed to detect a significant correlation between the memory B cell estimates obtained with DNA methylation- and transcriptome-based deconvolution, while the results from both analyses were consistent for most cell types [31]. Therefore, the cell proportion estimates derived from different types of data are not necessarily similar due to differences in the nature of the nucleic acids, the assay platforms, the data analysis pipelines, as well as other variables.

Our findings regarding the ECs are consistent with those of Aran et al. (2017) study, which showed that these cells are usually depleted in tumors compared to normal adjacent tissues [32]. The authors also reported that stromal cells are augmented in normal adjacent tissues, coinciding with wound response pathways enrichment. Normal adjacent tissue undergoes extracellular matrix remodeling, wound healing-like processes, fibrosis, and an epithelial-to-mesenchymal transition [33]. The explanation for these findings might depend on field cancerization and microenvironment effects. Field cancerization refers to pro-tumorigenic genetic mutations or epimutations that accumulate in cells within the pre-malignant field [34]. These cells are primed for cancer development and may not exhibit morphological changes. The microenvironment theory suggests that each cellular component of the tumor microenvironment (tumor, immune, and stromal cells) can communicate through direct interaction or secreted mediators, promoting aberrant signaling and enabling cancer initiation and progression [35]. In breast cancer, several studies have drawn attention to the fact that surrounding normal tissues, although histologically normal, are enriched with molecularly altered cells, which create an environment favorable to cancer progression [36].

The IBC and SNT composition comparison revealed that ECs exhibited the most significant divergence among the deconvolved cell types. ECs can modulate tissue and lymph node inflammation by interacting with and recruiting innate and adaptive immune cells [26]. They also function as non-hematopoietic antigen-presenting cells (APCs) since they can express class I and II major histocompatibility complex (MHC) proteins constitutively and at enhanced levels in response to inflammatory cytokines [37]. Although ECs lack the expression of co-stimulators CD80 and CD86, which are critical for activating naïve lymphocytes, they can mediate antigen-specific stimulation of effector and memory CD4^+^ and CD8^+^ T-lymphocytes [37].

We identified several EC surface proteins and mediators associated with hypermethylated CpGs by comparing IBC *versus* SNT. Cancer cells often undergo DNA demethylation globally, while hypermethylation occurs in promoters and enhancers in a cancer cell-specific manner, leading to transcriptional silencing [38]. The hypermethylated pattern identified in the promoters of EC markers *CD34*, *EMCN*, and *TEK* suggests a downregulated expression in tumor tissues. Still, according to the external cDNA microarray data, only *EMCN* exhibited a trend of downregulation in IBC compared to normal samples. It would be ideal to integrate gene expression and DNA methylation data generated with the same cohort of cases to confirm the correlation between promoter methylation and gene silencing for these markers.

*CD34* and *EMCN* are expressed by ECs on high endothelial venules (HEVs) and encode sialomucin proteins, which can bind adhesion molecule L-selectin and promote leukocyte rolling and transendothelial migration across HEVs [39, 40]. In non-lymphoid tissues, HEV-like blood vessels specialize in recruiting T- and B-lymphocytes during chronic inflammation and cancer [41]. A high density of HEVs within tumor stroma is associated with higher infiltration of B- and T-cells, including activated effector, naïve, and central memory T-cells, besides predicting longer survival rates in breast cancer [42]. These findings can explain the better survival observed in IBC patients with high proportion of ECs. Additionally, EC estimated proportions were positively correlated with memory B and memory CD8^+^ T infiltration in IBC. A recent study based on a CIBERSORT analysis of IBC versus non-IBC samples emphasized the IBC microenvironment heterogeneity, marked by M1 macrophages, γδ T-cells, and memory B cells [18]. The subpopulation of tissue-resident memory T cells knowingly exerts a peripheral immunosurveillance role and has been implicated in increased response to immune checkpoint inhibitors [43]. These interactions demonstrate the crucial role of chronic inflammation in IBC progression and the potential of targeting inflammatory pathways as a therapeutic strategy.

In the validation cohort, we demonstrated a significantly higher expression of CD34 in IBC compared to non-IBC samples. Accordingly, a previous immunohistochemistry study demonstrated that the number of ECs in IBC was at least eight times greater than in non-IBC specimens [44]. CD34^+^ ECs are described to migrate to lymph nodes and contralateral nascent breast cancer lesions to constitute new vessels, fueling angiogenesis, tumor progression, and metastases [45]. The angiogenic process is amplified by hypoxia-inducible factors, which trigger the release of proinflammatory mediators and the accumulation of stromal cells in breast cancer [46]. Also, the immunofluorescence results revealed significantly higher expression of CD68 in IBC than in the non-IBC samples, which reinforces the concept that macrophages release pro-angiogenic factors to drive breast cancer progression in a feedback loop [47]. Earlier evidence showed that macrophages enhance migration via RhoC-GTPase signaling in IBC, suggesting these cells are active players in the TME [48]. Additionally, the crosstalk between mesenchymal stem cells and macrophages, especially the M2 subtype, in the TME promotes IBC invasion and self-renewal [10]. As HiTIMED quantitatively assesses cell proportions but lacks spatial information, the immunofluorescence assays complemented its findings by providing spatial context by targeting cell-type-specific markers. Furthermore, HiTIMED does not have enough resolution to distinguish monocyte-lineage cells and their states, such as M1 and M2 phenotypes, which could help better elucidate the contribution of macrophages to the IBC microenvironment.

We analyzed a set of immune markers related to other cell types resolved by HiTIMED and potentially subjected to epigenetic silencing in IBC. Among them, *CD40* holds great promise for cancer immunotherapy. *CD40* is a member of the tumor necrosis factor (TNF) receptor superfamily expressed on APCs, including DCs and non-immune cells, such as platelets and ECs, and plays a role in adaptive immunity induction [49]. The interaction between CD40 on DCs and its ligand on CD4^+^ T cells promotes increased surface expression of MHC and costimulatory molecules, production of proinflammatory cytokines, and enhanced T-cell triggering by activated DCs [49]. Earlier evidence showed that *CD40* gene expression is significantly reduced in IBC compared with non-IBC samples, but we could not validate CD40 decreased expression at the protein level due to the scarcity of IBC samples [50]. According to our results, the promoter hypermethylation of *CD40* can lead to its reduced expression. DNA methylation levels were higher in IBC samples classified as immune cold, while immune hot samples presented intermediate methylation levels compared to SNT. The immune cold cluster was marked by reduced proportions of stromal cells, memory CD4^+^ T, CD8^+^ T and B-lymphocytes, DC, and NK cells. We also detected lower memory CD8^+^ T cell content in TN *versus* non-TN IBC. A recent preclinical study demonstrated that combining cytotoxic agents with CTLA4 inhibition and CD40 agonists may be more effective than targeting multiple checkpoints on T cells, especially in cold lesions as most TN breast tumors [51, 52]. CD40 agonism can produce tumoricidal myeloid cells in the absence of CD8^+^ T cell responses [49]. Therefore, modulating *CD40* expression with epigenetic drugs and targeting the gene product with CD40 agonists could improve therapeutic response even in tumors with poor CD8^+^ T cell infiltration.

Several agonistic anti-CD40 monoclonal antibodies (mAbs) have been developed and entered clinical trials, including CDX-1140, a human immunoglobulin G2 [53]. Two ongoing trials are evaluating the potential combination of CDX-1140 with other approaches to treat breast cancer (ClinicalTrials.gov IDs: NCT05029999 and NCT04616248). Although CD40 mAbs have not achieved substantial single-agent anti-tumor activity, except for melanoma, combination with chemotherapy, radiotherapy, or immunotherapy has resulted in tumor regression in different cancer types [54]. Epigenetic alterations are not restricted to cancer cells but also contribute to DC dysfunction in the TME. This is a limiting feature for anti-tumor immunity not addressed by immune checkpoint inhibitors. Targeting epimutations that affect different immune cell subsets should be considered as an option to enhance immunotherapy efficacy in IBC.

## Conclusion

DNA methylation-based deconvolution enabled us to characterize the IBC microenvironment and guided the search for dysregulated markers affected by underlying epigenetic changes. The complex interaction between the TME cellular components extends to the tumor surroundings, presenting higher immune and stromal infiltration than the tumor tissue. The EC component is notably reduced within the tumor stroma compared to its surroundings but seems to have anti-tumor properties in patients with higher EC infiltration. This is suggested by its favorable impact on IBC patients’ survival and positive correlation with immune cells, which are crucial for fighting back tumors. Additionally, previous evidence supports ECs contribution to innate and adaptive immune cell recruitment and antigen presentation [26]. The DC activity also seems deficient within TME since the methylation pattern of *CD40* promoter indicates that the CD40 surface molecule, fundamental for inducing adaptive immunity, is epigenetically silenced in IBC, especially in cold tumors. Therefore, insights gleaned from bulk DNA methylation data supported by preclinical and clinical evidence across various tumor types indicate that combining immunotherapy and epigenetic drugs is a viable alternative for treating inflammatory breast cancer.

### Electronic supplementary material

Below is the link to the electronic supplementary material.

Supplementary Material 1

Supplementary Material 2

Supplementary Material 3

Supplementary Material 4

Supplementary Material 5

Supplementary Material 6

Supplementary Material 7

Supplementary Material 8

## Data Availability

The dataset analyzed during the current study is available in the Gene Expression Omnibus (GEO) database (https://www.ncbi.nlm.nih.gov/geo/) under the accession number of GSE238092.

## Abbreviations

APCs: Antigen-presenting cells
DCs: Dendritic cells
DMPs: Differentially methylated probes
DMRs: Differentially methylated regions
ECs: Endothelial cells
ER: Estrogen receptor
FFPE: Formalin-fixed paraffin-embedded
GEO: Gene Expression Omnibus
HER2: Human epidermal growth factor receptor type 2
HEVs: High endothelial venules
HiTIMED: Hierarchical Tumor Immune Microenvironment Deconvolution
IBC: Inflammatory breast cancer
mAbs: Monoclonal antibodies
MHC: Major histocompatibility complex
NK: Natural killer cells
OS: Overall survival
PAM: Partitioning around medoids
PARPi: Poly (ADP-ribose) polymerase-1 inhibitor
PBS: Phosphate-buffered saline
Poly-ICLC: Polyinosinic-polycytidylic acid
PR: Progesterone receptor
SNT: Surrounding non-tumoral tissue
TME: Tumor microenvironment
TN: Triple negative
TNF: Tumor necrosis factor
