# Supplementary material for: Inflammatory breast cancer microenvironment repertoire based on DNA methylation data deconvolution reveals actionable targets to enhance the treatment efficacy
Source: J Transl Med. 2024 Aug 5;22:735. doi: 10.1186/s12967-024-05553-5 (PMC11301973; doi:10.1186/s12967-024-05553-5)
Supplement: Supplementary file 1 — Supplementary Material 1 [file 12967_2024_5553_MOESM1_ESM.docx]

**Supplementary Methods**

Genomic DNA was extracted from 24 inflammatory breast cancer (IBC) and surrounding non-tumoral tissue (SNT) samples and bisulfite-converted using the EZ DNA Methylation-Gold™ Kit (Zymo Research, Irvine, CA, USA). Genome-wide DNA methylation data was obtained with the Infinium Human MethylationEPIC BeadChip array (Illumina, San Diego, CA, USA). Data preprocessing, quality assessment, and normalization by Beta MIxture Quantile dilation (BMIQ) method [1] were performed using minfi R package (v.1.42.0) [2], while batch effects were removed using the ComBat function from sva R package (v.3.44.0) [3]. Differential methylation analysis was performed by comparing IBC and SNT samples using limma R package (v.3.50.3) [4]. Differentially methylated probes (DMPs) were identified considering false discovery rate (FDR) < 0.05 and |∆β|≥ 0.2. Differentially methylated regions (DMR) were determined using the DMRcate R package (v.2.10.0) [5]. Genomic regions with at least two consecutive DMPs (maximum gap of 1000 bp between two nearby CpG sites) and smoothed FDR < 0.05 were considered statistically significant. The DNA methylation analysis is described in detail by Faldoni et al. (2024) [6].

**Supplementary Tables**

**Table S1.** Clinical and pathological characteristics of 24 inflammatory breast cancer (IBC) patients from the discovery cohort and 17 IBC and 16 non-IBC patients from the validation cohort.

| **Features** | **Discovery set**  **IBC cases**  **N (%)** | **Validation set**  **IBC cases**  **N (%)** | **Validation set**  **Non-IBC**  **N (%)** | ***P-value^#^*** |
| --- | --- | --- | --- | --- |
| **Age (years**) |  |  |  |  |
| ≤ 50 | 11 (46) | 7 (41) | 8 (50) | 0.8783 |
| > 50 | 13 (54) | 10 (59) | 8 (50) |  |
| **Family history of cancer** |  |  |  |  |
| Unknown | 2 (8) | 2 (12) | 7 (43) | 0.1406 |
| No | 9 (38) | 8 (47) | 3 (19) |  |
| Yes | 13 (54) | 7 (41) | 6 (38) |  |
| Breast and/or ovarian cancer | 7 (54) | 5 (41) | 2 (38) |  |
| **Body Mass Index (Kg/m^2^)** |  |  |  |  |
| Normal (≤24.9) | 4 (17) | 5 (29) | 5 (31) | 0.5229 |
| Overweight (25 to 30) | 9 (38) | 7 (41) | 8 (50) |  |
| Obese (>30) | 8 (33) | 4 (24) | 1 (6) |  |
| Unknown | 3 (12) | 1 (6) | 2 (13) |  |
| **Histological grade^*^** |  |  |  |  |
| I | 1 (4) | 2 (12) | 2 (12) | 0.8301 |
| II | 15 (63) | 9 (53) | 8 (50) |  |
| III | 8 (33) | 6 (35) | 6 (38) |  |
| **Clinical stage** |  |  |  |  |
| III | 15 (62) | 8 (47) | 7 (44) | 0.4370 |
| IV | 9 (38) | 9 (53) | 9 (56) |  |
| **Hormone receptor (ER or PR) /HER2 status** |  |  |  |  |
| + /+ | 0 | 2 (12) | 2 (13) | **0.0058** |
| +/ - | 10 (42) | 14 (82) | 10 (62) |  |
| -/ + | 3 (12) | 0 (0) | 2 (13) |  |
| TN | 11 (46) | 1 (6) | 2 (13) |  |
| **Distant metastasis** |  |  |  |  |
| No | 7 (29) | 8 (47) | 6 (38) | 0.5032 |
| Yes | 17 (71) | 9 (53) | 10 (62) |  |
| At diagnosis (Stage IV) | 9 (37) | 4 (24) | 7 (43) |  |
| During follow-up | 8 (34) | 5 (29) | 3 (19) |  |
| **Survival status** |  |  |  |  |
| Alive | 5 (21) | 6 (35) | 6 (37) | 0.4448 |
| Dead | 19 (79) | 11 (65) | 10 (63) |  |

(+): positive; (-): negative; ER: estrogen receptor; PR: progesterone receptor; TN: triple-negative breast cancer; *according to Scarf-Bloom-Richardson grading system; ^#^The chi-square test with Yates’ correction or Fisher’s exact test.

**Table S4.** Univariate analysis of clinicopathological variables and estimated cell fractions using Cox regression.

| **Variable** | **Mean (SD)** | **Hazard ratio (CI)** | ***p*-value^#^** |
| --- | --- | --- | --- |
| Age (years) | 54.5 (13.7) | 0.98 (0.95-1.02) | *p* = 0.366 |
| Clinical stage |  |  |  |
| IIIB+IIIC | 15 (62.5) | - | - |
| IV | 9 (37.5) | 8.19 (2.60-25.78) | ***p* <0.001** |
| SBR grade |  |  |  |
| 1 | 1 (4.2) | - | - |
| 2 | 15 (62.5) | 1.91 (0.22-16.53) | *p* = 0.556 |
| 3 | 8 (33.3) | 3.04 (0.32-28.41) | *p* = 0.330 |
| Distant metastasis |  |  |  |
| Presence | 15 (65.2) | 15.76 (2.00-124.01) | ***p* = 0.009** |
| Absence | 8 (34.8) | - | - |
| M stage |  |  |  |
| M0 | 15 (62.5) | - | - |
| M1 | 9 (37.5) | 8.19 (2.60-25.78) | ***p* <0.001** |
| N stage |  |  |  |
| N0 | 1 (4.2) | - | - |
| N1 | 8 (33.3) | 181836127.05 (0.00-Inf) | *p* = 0.999 |
| N2 | 6 (25.0) | 1336733349.93 (0.00-Inf) | *p* = 0.998 |
| N3 | 9 (37.5) | 1221704532.87 (0.00-Inf) | *p* = 0.998 |
| *TP53* mutation* |  |  |  |
| Positive | 10 (41.7) | 1.82 (0.70-4.72) | *p* = 0.215 |
| Negative | 14 (58.3) | - | - |
| HRR genes mutation* |  |  |  |
| Positive | 7 (29.2) | 1.52 (0.54-4.29) | *p* = 0.427 |
| Negative | 17 (70.8) | - | - |
| Triple negative |  |  |  |
| Yes | 10 (41.7) | 2.32 (0.83-6.50) | *p* = 0.111 |
| No | 14 (58.3) | - | - |
| ER |  |  |  |
| Positive | 11 (45.8) | 0.49 (0.18-1.34) | *p* = 0.165 |
| Negative | 13 (54.2) | - | - |
| PR |  |  |  |
| Positive | 8 (33.3) | 0.90 (0.33-2.47) | *p* = 0.837 |
| Negative | 16 (66.7) | - | - |
| HER2 |  |  |  |
| Positive | 3 (12.5) | 0.91 (0.21-4.05) | *p* = 0.905 |
| Negative | 21 (87.5) | - | - |
| EC cluster |  |  |  |
| Low | 12 (50.0) | - | - |
| High | 12 (50.0) | 0.29 (0.10-0.87) | ***p* = 0.027** |
| Stromal cluster |  |  |  |
| Low | 12 (50.0) | - | - |
| High | 12 (50.0) | 0.33 (0.12-0.91) | ***p* = 0.033** |

CI: 95% confidence interval; SBR: Scarff-Bloom-Richardson grading system; HRR: homologous recombination repair; ER: estrogen receptor; PR: progesterone receptor; HER2: human epidermal growth factor receptor type 2; EC: endothelial cell; in bold: *p*-value <0.05. *Only pathogenic and likely pathogenic variants were considered. ^#^Wald test

**Table S5.** Kaplan-Meier survival analysis of the validation cohort based on clinicopathological variables and CD34 and CD68 protein expression.

| **Variable** | **Number of cases**  **(N=33)** | **Median survival (months)** | ***p*-value**  **(log-rank test)** |
| --- | --- | --- | --- |
| Age (years) |  |  |  |
| ≤55 | 18 | 33.50 | 0.6204 |
| >55 | 15 | 49.00 |  |
| Clinical stage |  |  |  |
| IIIB+ IIIC | 21 | - | **0.0138** |
| IV | 12 | 28.50 |  |
| SBR grade |  |  |  |
| 1+ 2 | 21 | - | **0.0138** |
| 3 | 12 | 28.50 |  |
| Distant metastasis |  |  |  |
| Presence | 20 | 29.00 | **0.0004** |
| Absence | 13 | - |  |
| M stage |  |  |  |
| M0 | 15 | 122.00 | **0.0304** |
| M1 | 11 | 28.00 |  |
| Mx | 7 | - |  |
| N stage |  |  |  |
| N0+ N1 | 15 | - | 0.0644 |
| N2+ N3 | 18 | 31.50 |  |
| Triple negative |  |  |  |
| Yes | 4 | 18.00 | **0.0067** |
| No | 29 | 49.00 |  |
| ER |  |  |  |
| Positive | 26 | 91.00 | **0.0046** |
| Negative | 7 | 19.00 |  |
| PR |  |  |  |
| Positive | 23 | 49.00 | 0.0808 |
| Negative | 10 | 33.50 |  |
| HER2 |  |  |  |
| Positive | 6 | 82.50 | 0.7928 |
| Negative | 27 | 37.00 |  |
| CD34 Expression |  |  |  |
| Low ($\leq$5.66%) | 17 | 38.00 | 0.7966 |
| High (>5.66%) | 16 | 40.50 |  |
| CD68 Expression |  |  |  |
| Low ($\leq$15.5%) | 19 | 43.00 | 0.1061 |
| High (>15.5%) | 12 | 92.50 |  |

SBR: Scarff-Bloom-Richardson grading system; ER: estrogen receptor; PR: progesterone receptor; HER2: human epidermal growth factor receptor type 2. In bold: *p*-value <0.05.

**References**

1. Teschendorff AE, Marabita F, Lechner M, Bartlett T, Tegner J, Gomez-Cabrero D, et al. A beta-mixture quantile normalization method for correcting probe design bias in Illumina Infinium 450 k DNA methylation data. Bioinformatics [Internet]. 2013;29:189–96. Available from: https://academic.oup.com/bioinformatics/article-lookup/doi/10.1093/bioinformatics/bts680

2. Aryee MJ, Jaffe AE, Corrada-Bravo H, Ladd-Acosta C, Feinberg AP, Hansen KD, et al. Minfi: a flexible and comprehensive Bioconductor package for the analysis of Infinium DNA methylation microarrays. Bioinformatics [Internet]. 2014;30:1363–9. Available from: https://academic.oup.com/bioinformatics/article-lookup/doi/10.1093/bioinformatics/btu049

3. Leek JT, Johnson WE, Parker HS, Jaffe AE, Storey JD. The sva package for removing batch effects and other unwanted variation in high-throughput experiments. Bioinformatics [Internet]. 2012;28:882–3. Available from: https://academic.oup.com/bioinformatics/article-lookup/doi/10.1093/bioinformatics/bts034

4. Ritchie ME, Phipson B, Wu D, Hu Y, Law CW, Shi W, et al. limma powers differential expression analyses for RNA-sequencing and microarray studies. Nucleic Acids Res. 2015;43:e47–e47.

5. Peters TJ, Buckley MJ, Chen Y, Smyth GK, Goodnow CC, Clark SJ. Calling differentially methylated regions from whole genome bisulphite sequencing with DMRcate. Nucleic Acids Res. 2021;49:e109–e109.

6. Faldoni FLC, Bizinelli D, Souza CP, Santana IVV, Marques MMC, Rainho CA, et al. DNA methylation profile of inflammatory breast cancer and its impact on prognosis and outcome. Clin Epigenetics [Internet]. 2024;16:89. Available from: http://www.ncbi.nlm.nih.gov/pubmed/38971778
