# Supplementary figures and images for: Inflammatory breast cancer microenvironment repertoire based on DNA methylation data deconvolution reveals actionable targets to enhance the treatment efficacy
Source: J Transl Med. 2024 Aug 5;22:735. doi: 10.1186/s12967-024-05553-5 (PMC11301973; doi:10.1186/s12967-024-05553-5)

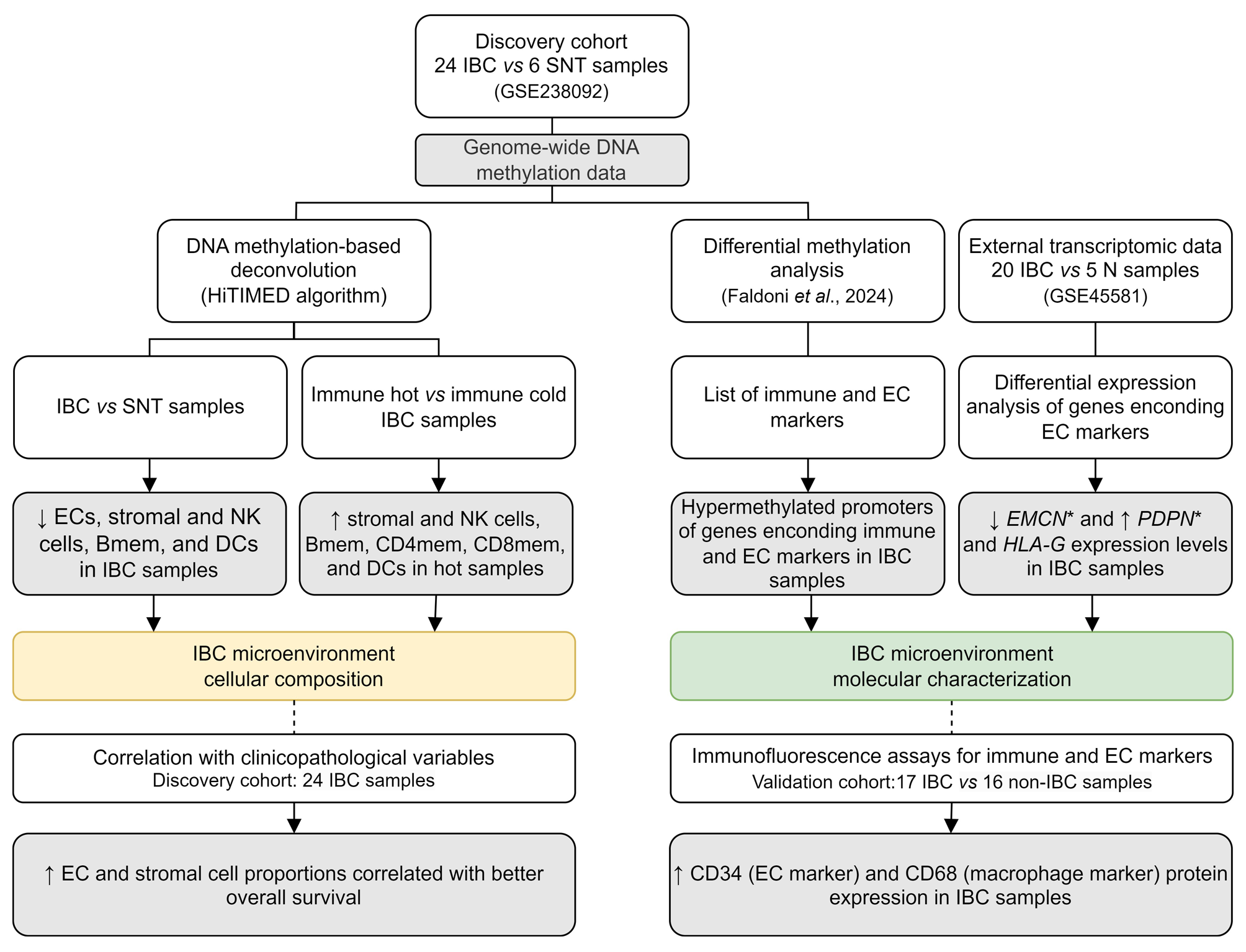

Supplement: Supplementary file 2 — Supplementary Material 2 [file 12967_2024_5553_MOESM2_ESM.tif]

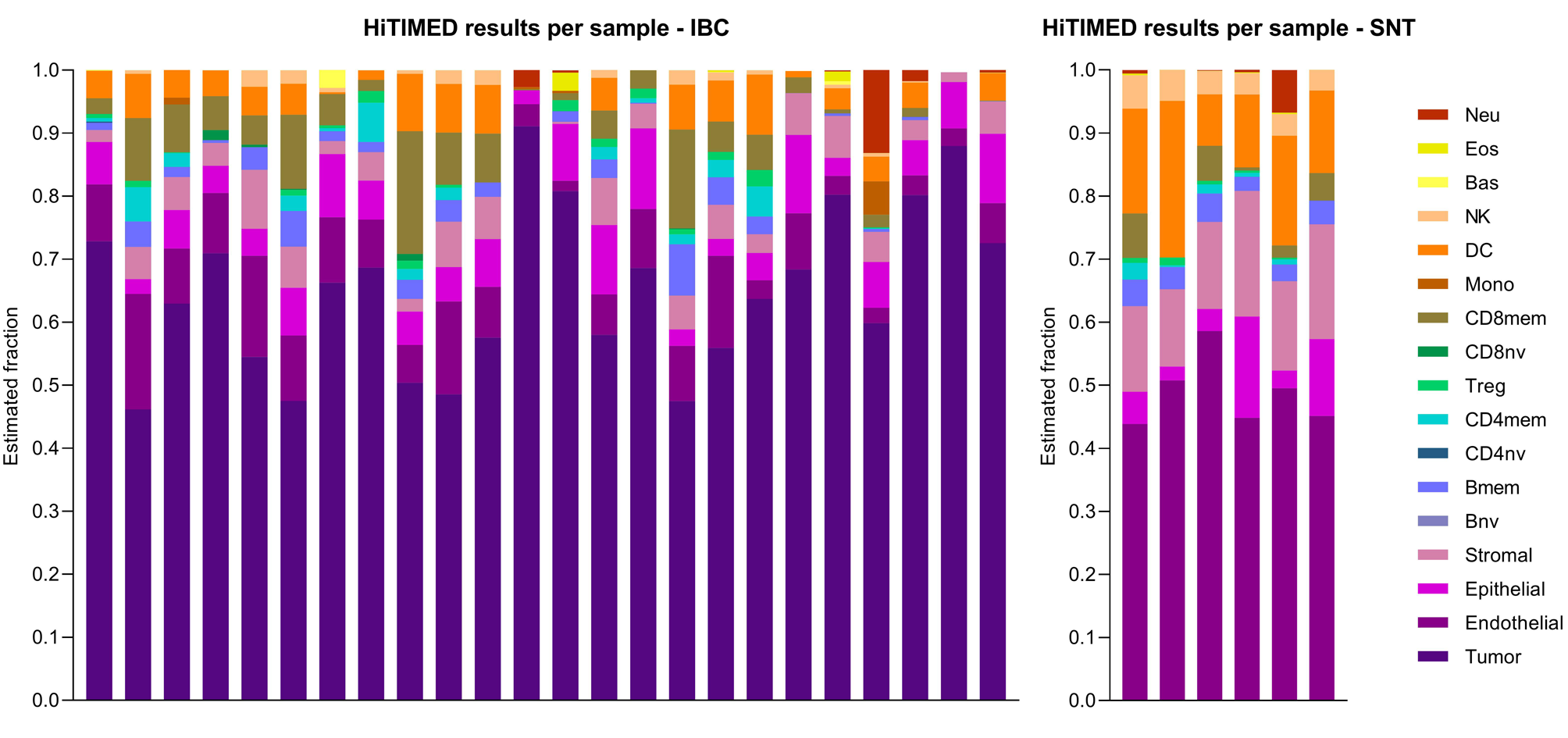

Supplement: Supplementary file 4 — Supplementary Material 4 [file 12967_2024_5553_MOESM4_ESM.tif]

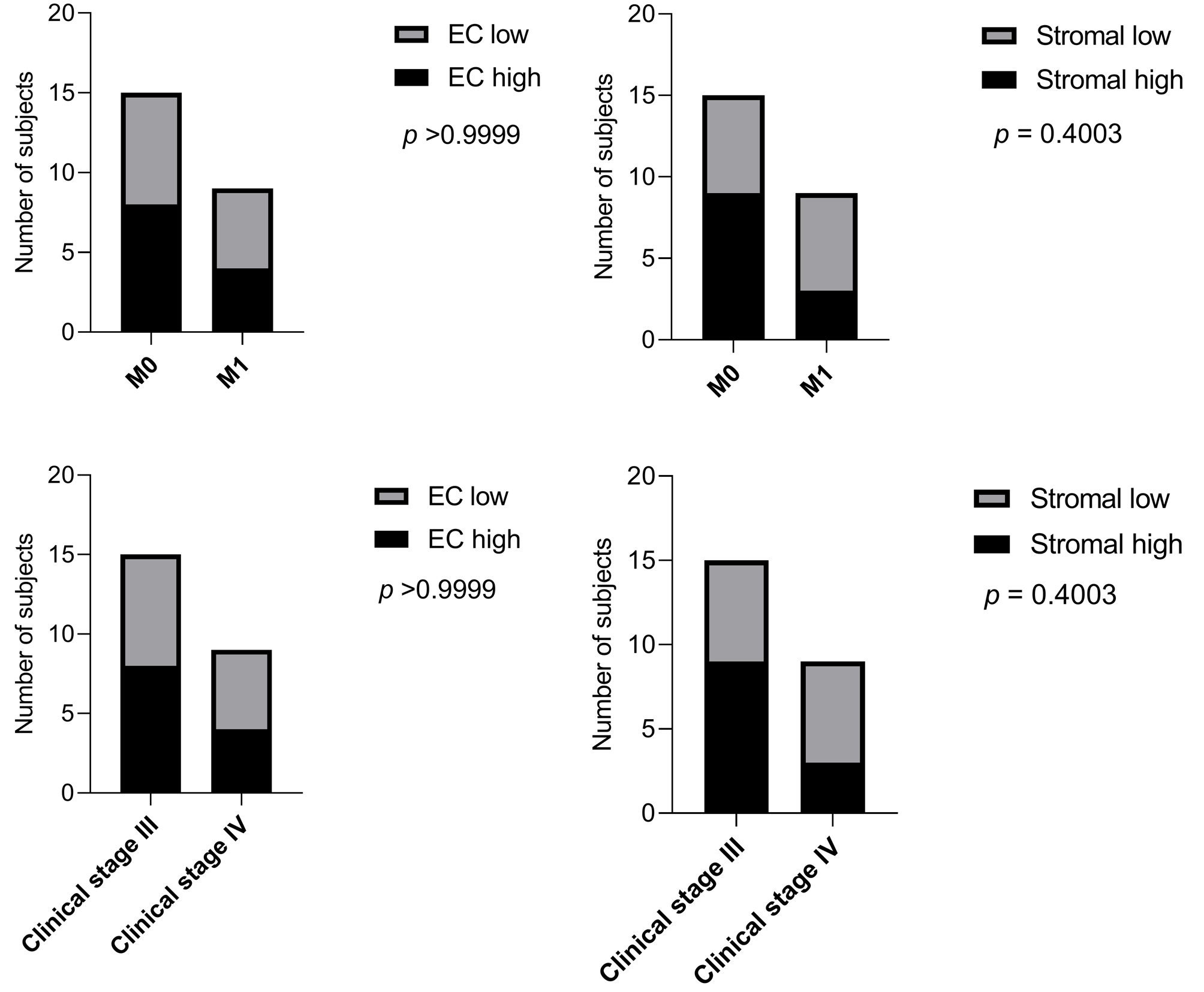

Supplement: Supplementary file 5 — Supplementary Material 5 [file 12967_2024_5553_MOESM5_ESM.tif]

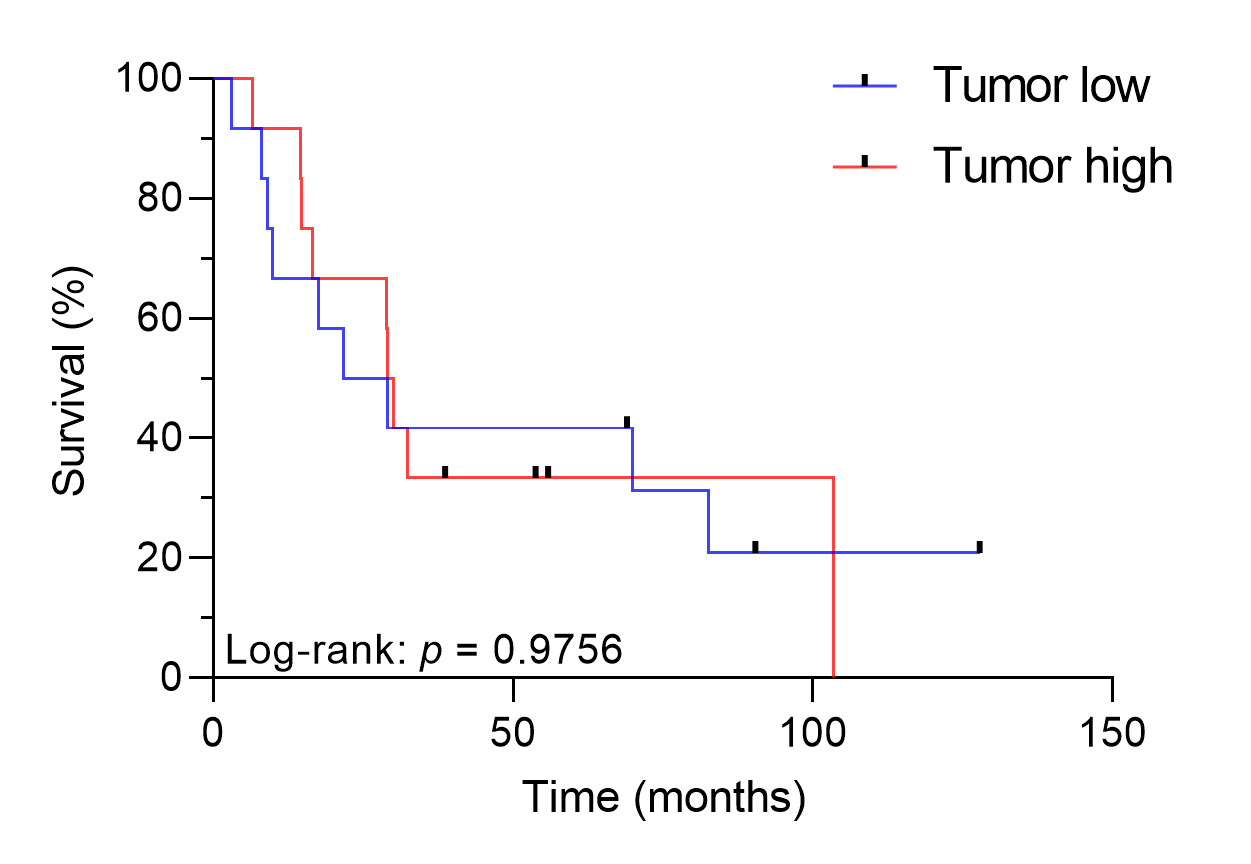

Supplement: Supplementary file 6 — Supplementary Material 6 [file 12967_2024_5553_MOESM6_ESM.tif]

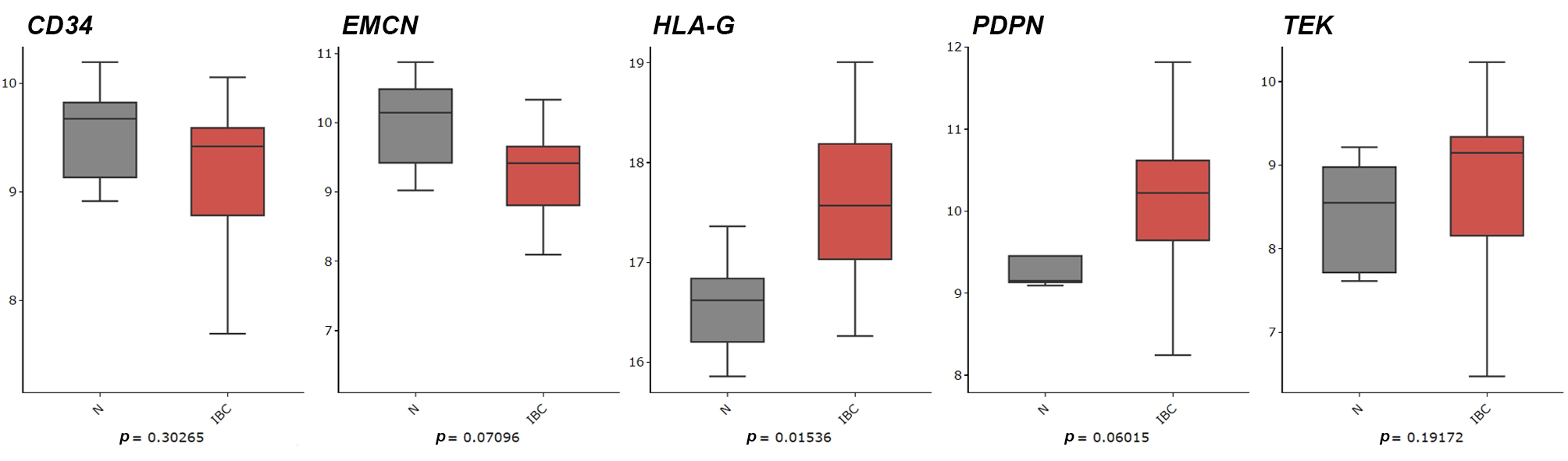

Supplement: Supplementary file 7 — Supplementary Material 7 [file 12967_2024_5553_MOESM7_ESM.tif]
