# Supplementary material for: Inflammatory breast cancer microenvironment repertoire based on DNA methylation data deconvolution reveals actionable targets to enhance the treatment efficacy
Source: J Transl Med. 2024 Aug 5;22:735. doi: 10.1186/s12967-024-05553-5 (PMC11301973; doi:10.1186/s12967-024-05553-5)
Supplement: Supplementary file 8 — Supplementary Material 8 [file 12967_2024_5553_MOESM8_ESM.pdf]

UNIVERSITY OF SOUTHERN DENMARK

Lillebaelt Hospital
University Hospital of Southern Denmark

Department of Clinical Genetics
University Hospital of Southern Denmark,
Institute of Regional Health Research
Beriderbakken 4, 7100 - Vejle - Denmark

July 18, 2024

To
Francesco M. Marincola, MD
Editor-in-Chief
Journal of Translational Medicine

Ref: Submission ID JTRM-D-24-05541

Dear Editor,

We want to thank you and the reviewers for all the comments and positive suggestions that helped us improve our manuscript. The answers and/or modifications are shown below, accompanied by specific comments. As recommended, the changes included in the manuscript are highlighted in gray.

All authors are aware of and agree to the content of this manuscript. The authors have no financial interest and no conflicts of interest to disclose. The manuscript or its essence has not been published, in whole or in part, and will not be submitted for publication elsewhere until the editor's final decision.

We look forward to hearing from you concerning the suitability of this manuscript for publication in the Journal of Translational Medicine.

Sincerely yours,

Silvia Regina Rogatto, Full Professor
Corresponding Author
Email: silvia.rogatto@unesp.br or silvia.regina.rogatto@rsyd.dk

POINT-BY-POINT RESPONSE TO GENERAL COMMENTS

Thank you for allowing us to submit the revised version of our manuscript. We also thank the reviewers for their comments and positive suggestions, which helped us improve our manuscript. The answers and/or modifications are shown below, accompanied by specific comments. The changes included in the manuscript are highlighted in gray.

Reviewer #1

This study investigates the tumor microenvironment of inflammatory breast cancer using DNA methylation data deconvolution. It identifies cellular compositions and epigenetically dysregulated markers with potential prognostic and therapeutic implications. While the findings are significant, several critical points need to be addressed to strengthen the manuscript.

1. Lines 105-106: Specify that you are conducting the study on mixed-stage IBC, and not only early stage, as might appear in these lines.

Answer: *In the Introduction section (lines 104-107), we stated the international consensus guidelines to treat most cases of inflammatory breast cancer (IBC) and locally advanced breast cancer (LABC). We included more information to clarify the sentence. We also updated the reference to present the most recent guidelines according to the Advanced Breast Cancer (ABC) Global Alliance (Cardoso F et al. Breast. 20248;76:103756. doi: 10.1016/j.breast.2024.103756). Our discovery cohort was composed only of patients with advanced breast cancers at clinical stages IIIB+IIIC (N=15) and IV (N=9) (detailed in Table S1). In this cohort, the patients received neoadjuvant chemotherapy (N = 13), neoadjuvant radiotherapy (N = 2), adjuvant chemotherapy (N = 1), adjuvant radiotherapy (N = 13), hormone therapy (N = 8), and palliative treatment (N = 13). Based on our main aims, the treatment strategies and their response were not included in the present study.*

2. Lines 118-120: Explain variations between IBC and Non-IBC: The differences in the compositions of immune and stromal cells between IBC and non-IBC should be elaborated. Additionally, it should be clear whether the data being referenced is preclinical or clinical.

Answer: *Our aim in this paragraph was to provide a brief review of tumor microenvironment components found in IBC. As recommended, we modified lines 120-130 to explain these pre-clinical and clinical studies.*

3. Cohort definition: Please, clearly define and denote the discovery cohort and the validation cohort. Include, patient stages, age, treatments, stages. Compare the two cohorts statistically in the distribution of these characteristics.

Answer: *We compared IBC samples from the discovery and validation cohorts following the reviewer's comments. The table below demonstrates that these cohorts were paired by age and clinical stage, M and N stage, and histological grade. Therefore, these findings indicated the coherence of evaluating the expression of CD34 and CD68 in the validation set as representative markers of the high endothelial and stromal cells identified in the discovery set. We also merged tables S1 and S2 (Additional file 1) to compare the discovery and validation cohorts showing clinical and histopathological similarities between these two cohorts. The manuscript was revised accordingly (lines 401-408; 506-508).*

Comparison of IBC groups from the discovery and validation cohorts.

	**IBC patients (Number of cases)**		
**Variable**	**Discovery cohort**	**Validation cohort**	***p*-value#**
Age (years)			
≤55	14	9	0.9814
>55	10	8	
Clinical stage			
IIIB+ IIIC	15	13	0.4986
IV	9	4	
M stage			
M0	15	13	0.4986
M1	9	4	
N stage			
N0+ N1	9	6	0.8535
N2+ N3	15	11	
Histological grade*			
I	1	2	0.8301
II	15	9	
III	8	6	

*According to Scarf-Bloom-Richardson grading system #The chi-square test with Yates' correction or Fisher's exact test.

4. Add in a schematic way also the experimental methods applied in each dataset and explain convincingly how the two methods are orthogonal for validation.

Answer: *We created a flowchart (Fig. S1, Additional file 2) depicting the experimental methods and analyses applied to each dataset of our study. The methods employed in the discovery and validation assays are orthogonal since they represent different experimental approaches to test the hypothesis generated. Hierarchical Tumor Immune Microenvironment Epigenetic Deconvolution (HiTIMED) is an algorithm used for high-resolution profiling of the tumor microenvironment by utilizing tumor-specific DNA methylation signatures to deconvolve cell types. It quantitatively assesses cell proportions but lacks spatial information. Remarkably, immunofluorescence can complement this gap by providing spatial context and confirming the presence and distribution of these specific cell types by targeting cell-type-specific markers. Therefore, the findings of the immunofluorescence assays for CD34 and CD68 cross-validated the accuracy of HiTIMED's cell type predictions of these two cell populations relevant in the context of our study. We included these comments in the Discussion section (lines 520-523).*

5. Lines 299-300 and stemming considerations: Survival Analysis, Univariate Effects and Cox Regression:
    a. Although a limited sample size precludes a meaningful multivariate analysis, it is essential to show univariate effects using Cox regression for all covariates. This analysis is needed to present the effect in terms of hazard ratio and could be supplemented with Wald's test instead of Cox regression.

Answer: *We appreciate the comments and suggestions. As recommended, we applied the univariate analysis of clinicopathological variables and estimated cell fractions associated with patients' survival using Cox regression. Next, we used the Wald test to determine the p-values. The results are included in the following table (Table S4). Among the six variables (clinical stage, distant metastasis, M stage, N stage, EC, and stromal cell proportions) that significantly impacted IBC patients' overall survival according to the Kaplan-Meier analysis presented in the manuscript (Table 1), five were also significant according to the Cox regression analysis: clinical stage, distant metastasis, M stage, EC, and stromal cell content. Both univariate analyses showed similar results. We added these findings to the manuscript (lines 324-329), and Table S4 was included in Additional file 1.*

Table S4. Univariate analysis of clinicopathological variables and estimated cell fractions using Cox regression.

Variable	Mean (SD)	Hazard ratio (CI)	*p*-value[#]
Age (years)	54.5 (13.7)	0.98 (0.95-1.02)	$p = 0.366$
Clinical stage			

IIIB+IIIC	15 (62.5)	-	-
IV	9 (37.5)	8.19 (2.60-25.78)	***p* <0.001**
SBR grade			
1	1 (4.2)	-	-
2	15 (62.5)	1.91 (0.22-16.53)	*p* = 0.556
3	8 (33.3)	3.04 (0.32-28.41)	*p* = 0.330
Distant metastasis			
Presence	15 (65.2)	15.76 (2.00-124.01)	***p* = 0.009**
Absence	8 (34.8)	-	-
M stage			
M0	15 (62.5)	-	-
M1	9 (37.5)	8.19 (2.60-25.78)	***p* <0.001**
N stage			
N0	1 (4.2)	-	-
N1	8 (33.3)	181836127.05 (0.00-Inf)	*p* = 0.999
N2	6 (25.0)	1336733349.93 (0.00-Inf)	*p* = 0.998
N3	9 (37.5)	1221704532.87 (0.00-Inf)	*p* = 0.998
TP53 mutation*			
Positive	10 (41.7)	1.82 (0.70-4.72)	*p* = 0.215
Negative	14 (58.3)	-	-
HRR genes mutation*			
Positive	7 (29.2)	1.52 (0.54-4.29)	*p* = 0.427
Negative	17 (70.8)	-	-
Triple negative			
Yes	10 (41.7)	2.32 (0.83-6.50)	*p* = 0.111
No	14 (58.3)	-	-
ER			
Positive	11 (45.8)	0.49 (0.18-1.34)	*p* = 0.165
Negative	13 (54.2)	-	-
PR			
Positive	8 (33.3)	0.90 (0.33-2.47)	*p* = 0.837
Negative	16 (66.7)	-	-
HER2			
Positive	3 (12.5)	0.91 (0.21-4.05)	*p* = 0.905
Negative	21 (87.5)	-	-
EC cluster			
Low	12 (50.0)	-	-
High	12 (50.0)	0.29 (0.10-0.87)	***p* = 0.027**
Stromal cluster			
Low	12 (50.0)	-	-
High	12 (50.0)	0.33 (0.12-0.91)	***p* = 0.033**

CI: 95% confidence interval; SBR: Scarff-Bloom-Richardson grading system; HRR: homologous recombination repair; ER: estrogen receptor; PR: progesterone receptor; HER2: human epidermal growth factor receptor type 2; EC: endothelial cell; in bold: *p*-value <0.05. *Only pathogenic and likely pathogenic variants were considered. [#]Wald test

b. Why not apply survival analysis also in the second cohort using CD34 and CD68? (since you discovered them in the first cohort, and validated expression in the second; why not validating survival? or at least try to?)

Answer: *In this new version of our manuscript, we performed survival analysis in the validation cohort comparing low and high CD34 and CD68 protein expression. The cutoff was based on the median value. Following the reviewer's recommendation, the results of this analysis are found in the new supplementary table S5 (Additional file 1). We found no statistically significant difference in overall survival based on the expression levels of CD34 and CD68. We included a paragraph in the Results section addressing these comments (lines 418-423).*

There are several potential explanations for this lack of statistical significance:

(1) ***Sample size:*** *The validation cohort was composed of 33 samples (17 IBC and 16 non-IBC), which can limit the statistical power to detect significant differences in survival. Considering the rarity of this tumor type, increasing the sample size was not possible to overcome this limitation. (2)* ***Cohort differences:*** *In the discovery cohort, a deconvolution analysis covered a myriad of cells present in bulk samples from inflammatory breast cancer (IBC) patients. The results of this analysis were correlated with clinicopathological variables and highlighted two cellular components: endothelial and stromal cells. To validate the findings, we select two markers, CD34 and CD68, each representing the cell components under analysis in the discovery cohort. The lack of statistical significance on survival between low and high marker expression might reside in the cell population profile within the tumor microenvironment of IBCs. Other specific endothelial and stromal markers to be determined could account for significant findings and influence survival outcomes. To consistently prove the prognostic value of these markers in all patient populations, larger sample sizes of this rare breast cancer are mandatory. (3)* ***Biological variability:*** *The biological behavior of IBC can vary among patients. The expression levels of CD34 and CD68 might be influenced by other underlying genetic, molecular, or environmental factors that were not analyzed in this study.*

In summary, the lack of statistical significance in survival analysis for CD34 and CD68 in the validation dataset likely arises from a combination of factors, including sample size, biological differences, and marker specificity for this type of cancer. Future research should delve deeper into these factors and uncover new insights.

c. The study population is heterogeneous regarding critical characteristics such as stage. This heterogeneity impacts the validity of survival analyses. To address this issue, consider two main options: Conduct separate analyses for non-metastatic and metastatic populations.

Check the distribution of the endothelial cell (EC) clusters and stromal clusters (high versus low) based on clinical stage (IIIB+IIIC versus stage IV) using Fisher's exact test. Verify the correlation between EC clusters and stromal clusters to determine if they are independent perspectives of the same biological effect.

Impact on Survival and Cluster Distribution: If significant differences are found in cluster distribution between non-metastatic and metastatic patients, it may affect the statements regarding survival impact. If Fisher's test shows significant differences in cluster distribution between non-metastatic and metastatic patients, refrain from making causal statements about the impact on survival. Instead, frame the results to indicate that metastatic patients have less stroma and suggest further studies to verify the impact on survival.

Answer: *As suggested, we verified the distribution of endothelial cell (EC) and stromal cell clusters (high versus low) according to the presence of metastasis (M0 versus M1) and clinical stage (IIIB+IIIC versus stage IV) using Fisher's exact test. The following stacked bar graphs depict the distribution of the clusters. Since we did not find statistically significant differences between non-metastatic and metastatic patients or stages III and IV (lines 319-323; Fig. S3, Additional file 5), we did not modify the manuscript regarding the EC and stromal cell content and the impact on IBC patients' survival.*

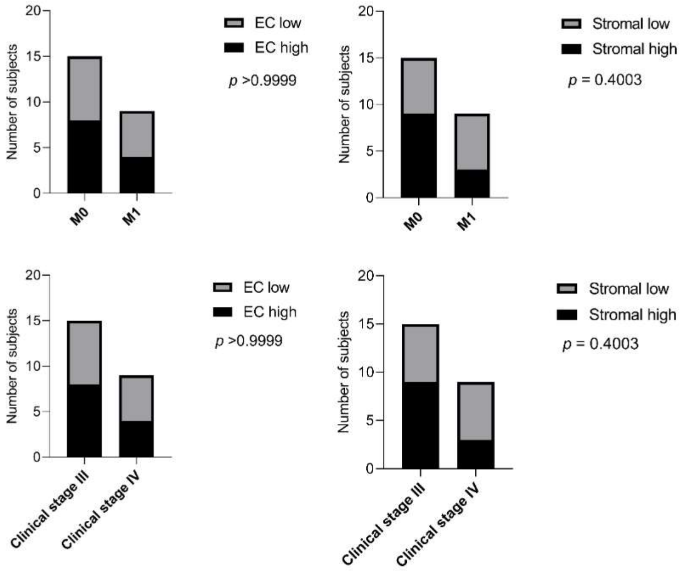

6. Validation of CD40 deregulation: The deregulation of CD40 identified in the discovery dataset was not explicitly validated in the second dataset. Include experiments to validate the CD40

findings in the second dataset or discuss this limitation clearly in the manuscript to provide transparency about the scope of the validation.

Answer: *We agree with the reviewer's suggestion that CD40 validation would strengthen our results. Regrettably, we are unable to proceed with this analysis due to the unavailability of additional FFPE tissue samples. IBC is a rare tumor, and it will be a challenge to get additional samples with the stringent criteria we adopted. It is imperative to conduct further studies in a different cohort of cases to augment our data. We have acknowledged this limitation of our study in the Discussion section (lines 533-536).*

Comments: The manuscript presents valuable insights into the IBC microenvironment and potential therapeutic targets. However, addressing the above points through major revisions will enhance the rigor and clarity of the study. By specifying the study focus, elaborating on key differences, conducting necessary statistical analyses, ensuring population homogeneity, and validating findings comprehensively, the manuscript will be significantly strengthened.

Answer: *Your valuable suggestions are greatly appreciated. Based on your comments, we modified the manuscript.*

Additional Recommendations:

Consider including a more detailed methodology section for the validation dataset to ensure reproducibility. Provide supplementary material with detailed statistical analyses and raw data to support the findings.

Answer: *As recommended, we detailed the CD34 and CD68 immunofluorescence assays performed in the validation cohort (lines 224-250). All the details and tables resulting from the DNA methylation data analysis are available in our open-access article recently published in Clinical Epigenetics (Faldoni et al. Clin Epigenetics. 2024, 16(1):89. doi: 10.1186/s13148-024-01695-x). The raw DNA methylation data was deposited in the Gene Expression Omnibus (GEO) database (https://www.ncbi.nlm.nih.gov/geo/) under the accession number GSE238092 and will be publicly available after our study's publication.*

Reviewer #2:

The authors conducted a microenvironment deconvolution analysis using methylation data from inflammatory breast cancer (IBC) and surrounding non-tumoral tissue (SNT). They identified patterns of cell infiltration in IBC and discovered a signature of CpG probes capable of classifying IBC tissues as immune hot or immune cold.

1. Although the paper presents interesting principles, it relies solely on a single tool, HiTIMED. It would be more robust if at least one additional deconvolution tool were used to validate the data.

Answer: *The HiTIMED developers compared its performance in deconvolving tumor microenvironment with two other methylation-based tools, MethylCIBERSORT and MethylResolver (Zhang Z et al. J Transl Med. 2022 Nov 8;20(1):516. doi: 10.1186/s12967-022-03736-6). HiTIMED showed the best performance in estimating the immune cell types from artificial mixture samples considering the mean absolute error. HiTIMED encompasses the 12 immune cell subsets captured by MethylCIBERSORT and includes five additional unique cell types, such as dendritic cells, which are prominent in shaping inflammatory breast cancer (IBC) microenvironment behavior (Chakravarthy A et al. Nat Commun. 2018 Aug 13;9(1):3220. doi: 10.1038/s41467-018-05570-1; Lim B et al. Nat Rev Cancer. 2018 Aug;18(8):485-499. doi: 10.1038/s41568-018-0010-y). Another advantage of HiTIMED is that the algorithm uses tumor-specific signatures derived from primary breast cancer cells. MethylCIBERSORT and MethylResolver trained their models on cancer cell lines, which might interfere with deconvolution accuracy since cancer cell lines harbor additional epigenetic alterations compared to primary tumors. MethylResolver also uses a universal standard reference for tumor purity estimation in all tumor types instead of organ-specific epithelial cell type DNA methylation signatures.*

As suggested, we used MethylCIBERSORT in IBC samples of the discovery cohort as an alternative method to deconvolve methylation data and compare it with HiTIMED results. The relative fractions for the 12 cell types estimated by both methods are depicted in the figure below. Due to the aforementioned limitations, the results obtained with HiTIMED are not necessarily reproducible using another methylation-based deconvolution method. This is why we did not include the results obtained with MethylCIBERSORT in the manuscript and chose to discuss the HiTIMED results.

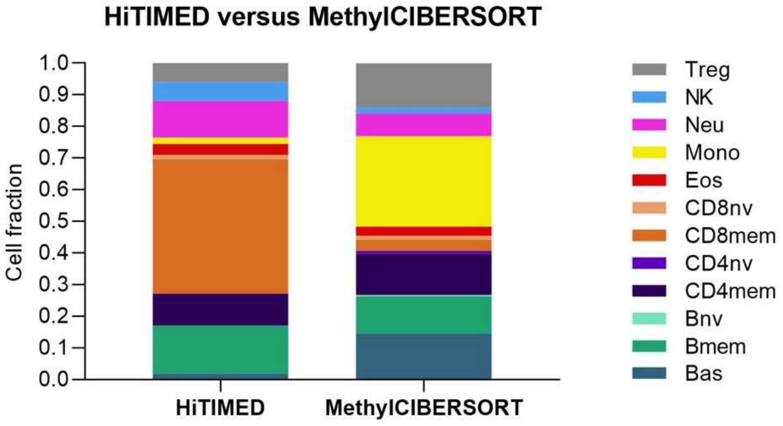

2. The study is limited to the authors' cohort, and the results need further validation for consistency using publicly available data.

Answer: *Inflammatory breast cancer is a rare disease (2-4% of all breast cancers) (Lim et al., Nature Rev Cancer 18: 485, 2018, DOI https://doi.org/10.1038/s41568-018-0010-y), which in a way justifies the sample number used in our study. Between 2002 and 2019, 140 IBC patients were admitted to Barretos Cancer Hospital, São Paulo, Brazil. From this large cohort, we only included biopsies obtained prior to chemo-radiotherapy treatment. A total of 116 available biopsies were re-analyzed by an expert pathologist. This analysis resulted in 24 fresh frozen IBC tissues included in the DNA methylation analysis (discovery set used for deconvolution analysis). Our cases were selected using stringent criteria and methods, including histopathological re-analysis and tissue microdissection. Although in a limited cohort of cases, our study shed light on understanding the microenvironment and the immune cell types associated with IBC.*

We fully share the reviewer's concern in validating our findings using publicly available data. However, due to the impossibility of obtaining the raw data described by der Auwera et al. (Van der Auwera I et al. PLoS One. 2010;5(9):e12616. doi: 10.1371/journal.pone.0012616), a direct comparison with IBC samples using the same parameters applied in our study was not feasible. We also recognize that methodological changes, particularly on the Illumina 27k platform used by these authors, might introduce variations in the coverage of CpG islands compared to the 850K Methylation platform used in our study. Further DNA methylation studies are needed to expand our knowledge of the biological and molecular mechanisms involved in IBC cases.

The paper provides data commentary without offering deeper insights; the authors need to add more comments. The authors are required to address the following issues:

3. Could you please provide the percentage of tumor fraction detected by HITIMED in the not-tumor tissues (SNT)? In Fig. 1b, this control is not represented. Would it be possible to add a supplementary figure showing the results of each sample profiled, to demonstrate the homogeneity/heterogenous composition across the IBC and SNT? What is the percentage of tumor-called tissue in the SNTs? If the tool identifies tumor tissue in SNT, how would you comment?

Answer: *To perform the deconvolution analysis with HiTIMED, it is necessary to specify the sample origin, whether a tumor or not. This algorithm uses a hierarchical model to resolve cell type proportions, and the first layer of deconvolution distinguishes tumor from nontumor cells (Zhang Z et al. J Transl Med. 2022 Nov 8;20(1):516. doi: 10.1186/s12967-022-03736-6). In the first layer, the tumor purity estimations are based on tumor-specific DNA methylation signatures. If not tumor, the algorithm function presets tumor purity to zero, as was the case for our surrounding non-tumoral tissue (SNT) samples. This is why the tumor fraction equals zero for SNT samples in Figure 1b.*

As recommended, we generated a supplementary figure (Fig. S2, Additional file 4) to demonstrate the heterogeneous cellular composition of IBC and SNT samples.

4. I'd like to ask for clarification regarding the input data used to perform the correlation in Fig. 2A. It seems that the authors conducted a correlation of detection values for all the samples for each pairwise comparison. The authors demonstrate a linear correlation between CD8Mem and Bmem. It would be beneficial to provide some explanatory comments on the highlighted data to aid the reader in interpreting the results. "Monocytes and neutrophils displayed inverse correlations with ECs" what are the implication of this result?

Answer: *The data inputted for the correlation analysis consisted of the cell fractions estimated by the HiTIMED deconvolution algorithm in IBC samples (N = 24). The pathogenesis and evolution of breast cancer depend on the tumor microenvironment, characterized by numerous inflammatory cells, including macrophages, monocytes, neutrophils, and lymphocytes (Di Bonito et al. Transl*

Cancer Res. 2019;8(Suppl 5):S449-S456. doi: 10.21037/tcr.2019.03.24; Gong et al. Pharmacol Res. 2023 Dec;198:106996. doi:10.1016/j.phrs.2023.106996). The present findings underscore a significant inverse correlation between monocytes, neutrophils, and endothelial cells, unveiling novel insights into the pathogenesis of IBC. The heightened levels of these inflammatory cells could be a critical factor in endothelial dysfunction. Monocytes may contribute to the aggressive nature of IBC by promoting angiogenesis and tumor cell intravasation. Notably, higher levels of inflammatory cytokines that modulate such mechanisms are associated with monocyte infiltration in IBC patients (Morrow et al. Mediators Inflamm. 2017; 2017:4754827. doi: 10.1155/2017/4754827; Mohamed et al. Int J Biochem Cell Biol. 2014; 46:138-47. doi: 10.1016/j.biocel.2013.11.015). Neutrophils also exacerbate endothelial damage by releasing reactive oxygen species, increasing neutrophil extracellular DNA traps (NETs), and facilitating the influx of tumor cells (McDowell et al. Nat Cancer. 2021;2(5):545-562. doi: 10.1038/s43018-021-00194-9). The current investigation also highlighted a linear correlation between CD8Mem and Bmem. It is in line with a previous study based on a CIBERSORT analysis of IBC versus non-IBC samples, which emphasized the IBC microenvironment heterogeneity, marked by M1 macrophages, γδ T-cells, and memory B-cells (Bertucci et al. Oncoimmunology. 2021;10(1):1929724. doi: 10.1080/2162402X.2021.1929724). The subpopulation of tissue-resident memory T cells knowingly exerts a peripheral immunosurveillance role and has been implicated with increased response to immune checkpoint inhibitors (Byrne et al. Nat Rev Clin Oncol. 2020;17(6):341-348. doi: 10.1038/s41571-020-0333-y). These interactions demonstrate the crucial role of chronic inflammation in IBC progression and the potential of targeting inflammatory pathways as a therapeutic strategy. Therefore, exploring the molecular mechanisms underlying these correlations and their implications for developing personalized treatment approaches for IBC patients is an unmet need.

As recommended, we expanded the Discussion section (lines 498-505) to clarify the interpretation of our results.

5. Fig 2B these results need to be validated by at least another dataset publicly available.

Answer: *We agree that showing the impact of endothelial and stromal cells on the survival of inflammatory breast cancer (IBC) patients from another cohort would strengthen our evidence. Van der Auwera et al. evaluated DNA methylation in IBC samples using the Infinium Human*

Methylation27 BeadChip. However, the raw data is not available for further comparison (Van der Auwera I et al. PLoS One. 2010 Sep 7;5(9):e12616. doi: 10.1371/journal.pone.0012616). To the Also, no other publicly available methylation dataset was generated with the same microarray platform (Infinium Methylation EPIC BeadChip) we used in IBC samples.

6. Did the authors perform differential analysis, or was it already available? If the authors performed the analysis, they should include the method in the write-up.

Answer: *The differential methylation analysis for the dataset used in our discovery cohort is detailed in a recently published study from our group (Faldoni FLC et al. Clin Epigenetics. 2024 Jul 6;16(1):89. doi: 10.1186/s13148-024-01695-x) included in the References section. We also briefly described DNA methylation data generation and analysis in Supplementary Methods (Additional file 1).*

7. Row 337: What do the authors mean by "normalized DNA methylation levels"? The method is not described. Did the authors correct for batch effects?

Answer: *To analyze the Infinium MethylationEPIC BeadChip array data, differences in Type-I and Type-II probes were adjusted, and β values were normalized using the Beta MIxture Quantile dilation (BMIQ) method (Teschendorff AE et al. Bioinformatics. 2013 Jan 15;29(2):189-96. doi: 10.1093/bioinformatics/bts680). The batch effect was corrected with the ComBat function from the sva package (v.3.44.0) (Leek JT et al. Bioinformatics. 2012; 28(6):882-3. doi: 10.1093/bioinformatics/bts034). The pipeline used for DNA methylation profiling was described in a recently published study from our group (Faldoni FLC et al. Clin Epigenetics. 2024;16(1):89. doi: 10.1186/s13148-024-01695-x) and summarized in Supplementary Methods (Additional file 1).*

8. Figure 4 shows the methylation probes of specific genes that support the concept of immune cold and immune hot environments. These probes can help classify IBC as hot or cold. Do these findings reflect at the transcriptomic level? Will the authors apply this concept to a larger dataset? The authors need to demonstrate the classification using PCR of those genes in matching tissues

Answer: *To classify inflammatory breast cancer (IBC) samples from the discovery cohort into immune hot or cold, we applied partitioning around medoids (PAM) clustering analysis to the cellular fractions estimated by the HiTIMED deconvolution algorithm. As far as we know, apart from a study by Van der Auwera et al. for which the raw data is not available (Van der Auwera I et al. PLoS One. 2010 Sep 7;5(9):e12616. doi: 10.1371/journal.pone.0012616), our dataset is the largest DNA methylation study derived from IBC samples. Additionally, HiTIMED cannot be used to perform transcriptome-based deconvolution, so we could not apply the same analysis pipeline to classify the IBC samples from the external gene expression dataset (GSE45581) included in our study.*

To further characterize the endothelial cell (EC) markers, we demonstrated the differences in DNA methylation between IBC and SNT samples (discovery cohort) and in gene expression between IBC and normal breast tissue samples (external transcriptomic data), as depicted by figures 4b and S4, respectively. The hypermethylated pattern identified in the promoters of EC markers CD34, EMCN, HLA-G, PDPN, and TEK suggests a downregulated expression in IBC tissues. However, according to the external cDNA microarray data, only EMCN exhibited a trend to downexpression, while HLA-G expression levels were increased, and PDPN presented a trend to overexpression in IBC compared to normal samples. Since the methylome and transcriptome profiles were derived from different cohorts, we consider that several variables, including clinicopathological characteristics and sample collection and processing, may preclude us from confirming the correlation between promoter hypermethylation and gene silencing for these markers. It would be ideal to integrate transcriptomic and DNA methylation data generated with the same cohort of cases to properly evaluate the correlation between promoter methylation and expression levels for EC and immune markers.

We used biopsies from IBC patients to perform the global DNA methylation analysis. The rarity of this disease and the limited amount of tissue available for molecular procedures prevents additional investigation. Unfortunately, we did not have RNA from fresh tissues from the discovery cohort or additional IBC cases to investigate the expression of genes encoding immune markers. Additionally, using formalin-fixed and paraffin-embedded (FFPE) samples for PCR is challenging. We opted to use FFPE tissues from biopsies of IBC patients of an additional cohort (validation set) to assess the immunophenotypes of CD34 and CD68 based on their association with IBC, as demonstrated in our data analysis.
